# Phylogenomic and metabolic insights into iron reduction metabolism in the genus Deferribacter belonging to the order Deferribacterales

**DOI:** 10.1099/mgen.0.001712

**Published:** 2026-05-19

**Authors:** Eva Pouder, Karine Alain, Sophie Mieszkin

**Affiliations:** 1 Univ Brest, Ifremer, CNRS, EMR 6002 BIOMEX, BEEP, F-29280 Plouzané, France

**Keywords:** *Deferribacter*, central metabolism, Fe(III)-reduction, *Deferribacterales*

## Abstract

Iron is one of the most important elements of the Earth, yet its bioavailability is limited in oceanic environments. In this context, deep-sea hydrothermal ecosystems represent one of the major sources of iron. While some microorganisms involved in its biogeochemical cycle, particularly in Fe(III)-reduction, have been isolated from these ecosystems, the molecular mechanisms underpinning metabolic pathways remain hypothetical and incomplete. Therefore, this study aims to investigate the global metabolism of bacteria within the *Deferribacter* genus, isolated from hydrothermal systems and a petroleum reservoir, with a specific focus on the Fe(III)-reduction metabolism to identify genes potentially involved in this pathway. This study revealed a conserved carbon metabolism across the four species, while their energetic metabolism exhibited notable differences. These species appear to be able to use different elements as electron sources, showing their ability to adapt to different ecological (micro)niches, particularly in deep-sea hydrothermal vents. The marker genes known for Fe(III)-reduction were identified, with a contrast between the strains isolated from hydrothermal systems and the one isolated from a petroleum reservoir. To further explore this pattern, the study was extended, including 14 genomes of representative strains and 36 metagenome-assembled genomes affiliated to the *Deferribacterales* order. Phylogenomic analysis revealed a distribution pattern within this order that correlates with environmental origin. Canonical marker genes of Fe(III)-reduction were also identified, with their distribution primarily aligned with specific ecological niches.

**Impact Statement**

Given that deep-sea hydrothermal ecosystems are one of the most important sources of iron in the oceans, it is essential to comprehensively understand the mechanisms governing iron in these environments, particularly the biotic processes such as Fe(III)-reduction. This study focuses on the metabolism of the four species belonging to the *Deferribacter* genus, mainly isolated from deep-sea hydrothermal vents and known for their ability to reduce Fe(III). The genomes of *Deferribacter abyssi* and *Deferribacter thermophilus* were newly sequenced and compared to the previously sequenced genomes of *Deferribacter autotrophicus* and *Deferribacter desulfuricans*. By integrating these new data with publicly available genome assemblies, this study expands our understanding of the genetic potential for carbon and energy metabolisms of the *Deferribacter* genus. In particular, investigations into the Fe(III)-reduction pathways in the *Deferribacter* genus, and more broadly within the *Deferribacterales* order, suggest that the distribution of Fe(III)-reducing capabilities is environment-dependent. Future transcriptomic investigations of *Deferribacter* species under Fe(III)-reduction and non-Fe(III)-reduction conditions could further elucidate the molecular basis of Fe(III) reduction in this group.

## Data Summary

Sequencing data and genome assemblies are available at GenBank as BioProject PRJNA906472 and PRJNA906486 and under the accession numbers GCA_049472655.1 and GCA_049472675.1 for *Deferribacter abyssi* and *Deferribacter thermophilus*, respectively. All supporting data and protocols are provided within the article and supplementary data files.

## Introduction

Iron (Fe) is one of the Earth’s most critical elements and an essential micronutrient for microbial growth. It can be used in assimilative metabolism, serving as a component of metalloproteins and enzyme cofactors, but also in energy metabolism as an electron donor (ferrous Fe, Fe(II)) or acceptor (ferric Fe, Fe(III)). While Fe is the fourth most abundant metal in the Earth’s crust [1], it is scarce in modern oceans. Marine hydrothermal vents, in a basaltic context, are an exception, as they are rich in Fe and make a major contribution to the Fe fertilization of oceans [2,5]. At deep-sea hydrothermal vents (DSHV), Fe is found in its two most important oxidation states: the oxidized form (Fe(III)) and the reduced form (Fe(II), sulphurized and non-sulphurized). In vent plumes, Fe(III) accounts for 60–70% of total Fe, while non-sulphurized Fe(II) accounts for 20–30% of total Fe, and around 10% of total Fe is represented by the sulphurized Fe(II) form [6]. Furthermore, in the distal part of hydrothermal plumes, the dominant forms of Fe are Fe(III) amorphous oxyhydroxides [7]. In the presence of oxygen at neutral pH, reduced and dissolved Fe(II) will spontaneously oxidize to Fe(III), which will react instantly with water to form poorly soluble iron oxyhydroxides [6]. Fe is thus poorly available for biological reactions but can be solubilized through chelation by siderophores and organic acids present in the environment, forming a soluble complex, enabling its uptake across the cell wall [8]. In addition, it was demonstrated by Li *et al*. [9] that Fe found in hydrothermal plumes and minerals can be used by microorganisms for energy production, contributing to its dispersion in the global ocean. Therefore, in this chemosynthesis-based ecosystem, at the boundary between ocean and lithosphere, Fe could play an important role in the biogeochemical cycles. While a major effort is currently being made to better identify the microorganisms involved in Fe(II)-oxidation and the metabolic pathways involved, the biotic Fe(III)-reduction in hydrothermal environments is still largely unknown.

Extracellular electron transfer (EET), such as in the Fe(III)-reducing process, occurs in Gram-negative and Gram-positive bacteria, as well as in archaea, and requires direct contact with minerals, often mediated by *c*-type cytochromes [10,14]. While Gram-positive bacteria have been reported to utilize polymeric substances for EET [13], most studies on Fe(III)-reducing bacterial strains focused on Gram-negative bacteria (*e.g*., *Geobacter* sp. and *Shewanella* sp.) originating from terrestrial or shallow marine environments. These microorganisms have evolved diverse strategies involving a variety of cytochromes. Among them, the Mtr and Omc pathways have been described and involve electron transfer from the inner to the outer membrane via the periplasm [15,16]. To date, the MtrCAB pathway, composed of *c*-type cytochromes (CymA, MtrA, MtrC and OmcA) and a porin-like transmembrane protein (MtrB), has been identified, but this pathway is still putative and incomplete [17]. The Omc pathway involved poly-haem *c*-type cytochromes OmbB and OmcC and the mono-haem *c*-type cytochrome OmbF or other cytochromes, such as OmcS, OmcZ or OmcB, for example [18,22]. However, the Omc pathway is also only partially deciphered and still unclear, notably concerning the electron transfer in the periplasm. Despite these advances in deciphering the Fe(III)-reduction pathways, it is obvious that they are not all elucidated so far, especially those used by bacteria from DSHV ecosystems. Recently, using metatranscriptomic analysis, the *mtrAB* gene complex has been used to identify Fe(III)-reducers in DSHV microbial mats [23]. However, although the involvement of this complex in Fe-reduction has been well established, these genes have not been identified in Fe(III)-reducing strains such as *Geothermobacter* sp. HR-1 [24] or *Deferribacter autotrophicus* [25].

To provide new insights into Fe(III) reduction metabolic pathways, this study presents a comparative genomic analysis of the four species belonging to the genus *Deferribacter* (order *Deferribacterales*). Among these four species, three have been isolated from DSHV ecosystems (*Deferribacter abyssi* JR^T^, *D. autotrophicus* SL50^T^ and *Deferribacter desulfuricans* SSM1^T^) and the fourth species from a petroleum reservoir (*Deferribacter thermophilus* BMA^T^) [26,29]. They encompass three Fe(III)-reducing species (*D. abyssi*, *D. autotrophicus* and *D. thermophilus*), which probably have a Fe(III)-reduction pathway other than the MtrCAB or Omc pathways. For this reason, the *Deferribacter* genus is an excellent model for studying the genetic potential of lineages likely to possess a new Fe(III)-reduction pathway. This study also examines genomes of cultivated representatives and metagenome-assembled genomes (MAGs) belonging to the *Deferribacterales* order with the aim of investigating the metabolic capabilities that may be associated with Fe(III)-reduction pathways within this order, in an attempt to identify environmental or phylogenetic patterns.

## Methods

### Genome sequencing and assembly

*D. abyssi* JR^T^ (=DSM 14873^T^) and *D. thermophilus* BMA^T^ (=DSM 14813^T^) strains were ordered from the DSMZ collection to sequence their genomes. Biomass production was carried out in the DSMZ 935A medium (*D. thermophilus* medium), respectively at 55 °C and 60 °C for *D. abyssi* JR^T^ and *D. thermophilus* BMA^T^. Cells were harvested from 75 ml of culture in the late exponential phase of growth, after 24 h of incubation. Genomic DNA was then extracted using the phenol/chloroform/isoamyl alcohol (PCI 25 : 24 : 1) method, as described previously [30], with the addition of 50 µM linear acrylamide to enhance nucleic acids precipitation (Invitrogen^™^). Quality of nucleic acids in solution was determined using the NanoDrop^™^ 8000 (Thermo Scientific, Waltham, MA, USA) spectrophotometer. Double-strand DNA concentrations were measured using the kit Quantifluor^™ ^dsDNA system (Promega^®^) according to the manufacturer’s instructions.

The whole-genome sequencing of the strains was performed by the Novogene company (Cambridge, UK), using the Illumina NovaSeq technology (2×150 bp paired reads, Micro Nano V2 chemistry). Read quality control and assembly were performed on the Galaxy France platform (https://usegalaxy.fr). Quality control was performed with FastQC (v0.73). Reads were then cleaned using the Trimmomatic (v0.38.1) tool with default parameters [31]. Genomes were assembled using reads from both before and after trimming and using both SPAdes (v3.15.4) and Shovill (v1.1.0) with default parameters [32,33]. Quast (v5.2.0) was used to assess the quality of genome assemblies [34] and to perform statistics to compare the different assemblies. The best assembly for each genome was selected for downstream annotation. The completeness and potential redundancies of each genome were assessed using BUSCO (v5.3.2) and CheckM (v1.2.3) [35] on the MicroScope Microbial Genome Annotation and Analysis Platform (MaGe) (https://mage.genoscope.cns.fr) [36] as well as with CheckM2 (v2.3.2) in the command line [37].

### Genome annotation and environmental distribution of the *Deferribacter* genus

To elucidate the global metabolism (carbon, nitrogen, sulphur, hydrogen and respiratory metabolism), genome assemblies were firstly annotated using different tools such as Prokka (v1.14.6) [38] on the Galaxy France platform [39] and using KEGG and BioCyc databases on the MaGe platform (https://mage.genoscope.cns.fr) [36]. Then, annotations were performed using the National Center for Biotechnology Information (NCBI Prokaryotic Genome Annotation Pipeline (PGAP) [40]. In addition, the eggNOG database was used to classify coding DNA sequences (CDS) into clusters of orthologous groups (COG) [41]. Genes involved in Fe metabolism were identified using FeGenie in command line (v1.2) according to Garber *et al*. [42], and their functional annotation was subsequently confirmed through manual curation on the MaGe platform, sequence alignments with the SwissProt database and comparative analysis via blast against the UniProt database [43,44]. Genome assemblies and annotations were compared to those of the two other species of the genus [*i.e*., *D. autotrophicus* SL50^T^ (GCA_008362905.1) and *D. desulfuricans* SSM1^T^ (GCA_000010985.1)]. To highlight the distribution of the *Deferribacter* genus across diverse ecosystems, the Sandpiper resource (v0.3.0) was used. This web interface allows identifying genomes or draft genomes in public shotgun metagenomic datasets on the basis of the analysis of highly conserved regions of single-copy marker genes through the use of the SingleM pipeline [45]. To do so, the search was performed at the *Deferribacter* genus level on the Sandpiper web-interface (https://sandpiper.qut.edu.au/taxonomy/g__Deferribacter) to obtain its relative abundance in public metagenomes linked to its environmental distribution.

### Dataset compilation

It has been shown that within the 14 species of the order *Deferribacterales* that have been isolated and characterized from a physiological point of view, seven of them are capable of heterotrophic or autotrophic growth by using ferric iron as a terminal electron acceptor for energy production. As these 14 strains were isolated from various environments, a phylogenomic analysis was performed at the level of the *Deferribacterales* order to investigate if there are any general patterns associating monophyletic groups with specific environments. Public genomes and MAGs from the order *Deferribacterales*, downloaded from NCBI, were also included in this phylogenomic analysis. Completeness and redundancy of these genomes and MAGs were assessed with CheckM2 (v1.0.1) [37]. Medium-quality (completeness >70% and redundancy <10%) and high-quality (completeness >90% and redundancy <5%) genomes and MAGs were retained for further analyses (Table S1, available in the online Supplementary Material) [46].

### Phylogenomic analysis

The phylogenomic tree of the *Deferribacterales* order was built using the Genome Taxonomy Database Toolkit (GTDB-tk) [v2.4.0 (release 220)] [47]. Briefly, the *identify* command line was used to identify the pool of single-copy core genes (SCGs) to use. Next, the alignment of the 120 bacterial SCGs was performed using the *align* command and trimAl (v1.4.1) [48] was used to remove gaps representing more than 50% of the nucleotide positions. Finally, the phylogenomic tree was reconstructed using the maximum-likelihood method with the IQ-TREE tool (v2.2.5) [49] with 1,000 ultra-fast bootstrap replicates. It was visualized using FigTree (v1.4.4) [50]. The genome of *Clostridium butyricum* CDC 51208 was used as an outgroup. The average nucleotide identity (ANI) scores and the average amino acid identity (AAI) scores between genomes and MAGs belonging to the *Deferribacterales* order were, respectively, obtained using the FastANI tool on the Galaxy France platform (v1.3) [51] and the EzAAI suite of workflows (v1.2.3) [52].

### Phylogenetic analysis

All PilA protein sequences identified using the FeGenie software or the MaGe platform were aligned using ClustalW (v2.1) [53] on the Galaxy France platform [39]. The phylogenetic tree was reconstructed using the maximum-likelihood method with the IQ-TREE tool (v2.2.5) [49] with 1,000 ultra-fast bootstrap replicates and visualized using FigTree (v1.4.4) [50]. The sequence of PilA detected in *Shewanella oneidensis* MR-1 was used as an outgroup. In addition, the PilA sequences of *D. abyssi*, *D. autotrophicus*, *Geobacter sulfureducens* and *Geobacter metallireducens* were aligned using ClustalW to analyse their aromatic amino acid (AAA) composition [phenylalanine (F), tyrosine (Y), tryptophan (W) and histidine (H)].

## Results and discussion

### General genomic features

For *D. abyssi* and *D. thermophilus*, the best assemblies were obtained with Shovill and SPAdes, respectively. The draft genomes obtained were composed of 43 contigs for *D. abyssi* and 31 contigs for *D. thermophilus*. All genome metrics are listed in Table 1. The overall genome sizes were 2,267,994 nucleotides with a GC content of 31.1 mol% and 2,195,820 nt with a GC content of 30.67 mol% for *D. abyssi* and *D. thermophilus*, respectively. These parameters are consistent with those of the chromosome of *D. desulfuricans* and slightly lower than those obtained for *D. autotrophicus* [25].

**Table 1.** Physiological characteristics and genomic features of the four species of *Deferribacter*

	*D. thermophilus* BMA^T^	*D. abyssi* JR^T^	*D. autotrophicus* SL50**^T^**	*D. desulfuricans* SSM1^T^ chromosome plasmid	
**Physiological features**					
**Origin**	Produced water from pretoleum reservoir	Deep-sea hydrothermal vent chimney (2,400 m)	Deep-sea hydrothermal vent chimney (4,100 m)	Deep-sea hydrothermal vent chimney (1,385 m)	
**Geographic location**	The Beatrice Oil Field (North Sea)	Rainbow (Atlantic Ocean)	Ashadze (Atlantic Ocean)	Suiyo Seamount (Pacific Ocean)	
**T°C range**	50–65 [60]	45–65 [60]	25–75 [60]	40–70 [60–65]	
**pH range**	5–8 [6.5]	6.0–7.2 [6.5]	5–7.5 [6.5]	5–7.5 [6.5]	
**(NaCl) range (g.l^−1^**)	0–50 [20]	10–50 [30]	10–60 [25]	18–96 [36]	
**Carbon sources and electron donors**	Pyruvate, malate, lactate, succinate, citrate, acetate/H_2_	Yeast extract, peptone, pyruvate, acetate, tryptone, succinate, CO_2_/H_2_	Yeast extract, peptone, pyruvate, acetate, succinate, formate, lactate, propionate, CO_2_/H_2_	Yeast extract, peptone, pyruvate, tryptone, formate, propionate, casein, acetate/H_2_	
**Respiratory type**	Anaerobic	Anaerobic	Anaerobic	Anaerobic	
**Electron acceptors**					
**Fe(III**)	+	+	+	–	
**S^0^**	–	+	+	+	
**Mn(IV**)	–	–	+	+	
**NO_3_**^–^	+	+	+	+	
**References**	[26]	[27]	[29]	[28]	
**Genomic features**					
**Number of contigs (>200 pb**)	31	43	10	1	1
**Largest contig**	386,288	230,198	319,692	2,234,389	308,544
**Total length (bp**)	2,195,820	2,267,994	2,543,880	2,234,389	308,544
**N50**	201,975	109,065	−	−	−
**%GC**	30.67	31.10	32.62	31.12	24.46
Completeness (%)*	99.99	99.66	99.91	99.93	
**Redundancy (%)** *	0.0	0.0	0.0	0.35	
**CDSs number**					
	2,233	2,299	2,451	2,117	
**Pseudogenes**	8	4	23	17	9
**RNA genes**	48	48	53	–	–
**rRNA operons**	1	1	2	2	–
**tRNA**	41	40	42	43	–
**Small ncRNA**	4	4	4	2	–
**References**	This study†	This study†	[25]	[63]	

*Completeness and redundancy were determined with CheckM2.

†The data were obtained from the MaGe platform and the QUAST tool.

–, data not available.

Annotations with PGAP resulted in the prediction of 2,288 genes, including 2,237 protein-coding genes, 1 RNA operon, 41 tRNAs, 4 ncRNAs and 3 pseudogenes for *D. abyssi*, and 2,181 genes, including 2,167 protein-coding genes, 1 RNA operon, 41 tRNAs, 4 ncRNAs and 14 pseudogenes for *D. thermophilus*. According to the EggNOG classification, genomes of *D. abyssi* and *D. thermophilus* encoded 2,299 and 2,233 CDS, respectively (Table 1). Most of the CDS, 92.34% (2,123/2,299) for *D. abyssi* and 94.85% (2,118/2,233) for *D. thermophilus*, were assigned to at least 1 COG category (Table S2). Among major processes, COGs categories related to metabolism were dominant (34.75% of the CDS for *D. abyssi* and 38.07% for *D. thermophilus*) and included notably inorganic ion transport and metabolism (4.31 and 5.28%). Then, 24.87% and 25.24% of the CDS were involved in cellular processes and signalling in *D. abyssi* and *D. thermophilus*, respectively. Finally, 17.13% of the COGs of *D. abyssi* and 16.83% of those of *D. thermophilus* were dedicated to information storage and processing. However, a significant part of these CDS had an unknown function, namely 20.92% in *D. abyssi* and 20.73% in *D. thermophilus*.

### Carbon metabolism

**Fig. 1.**
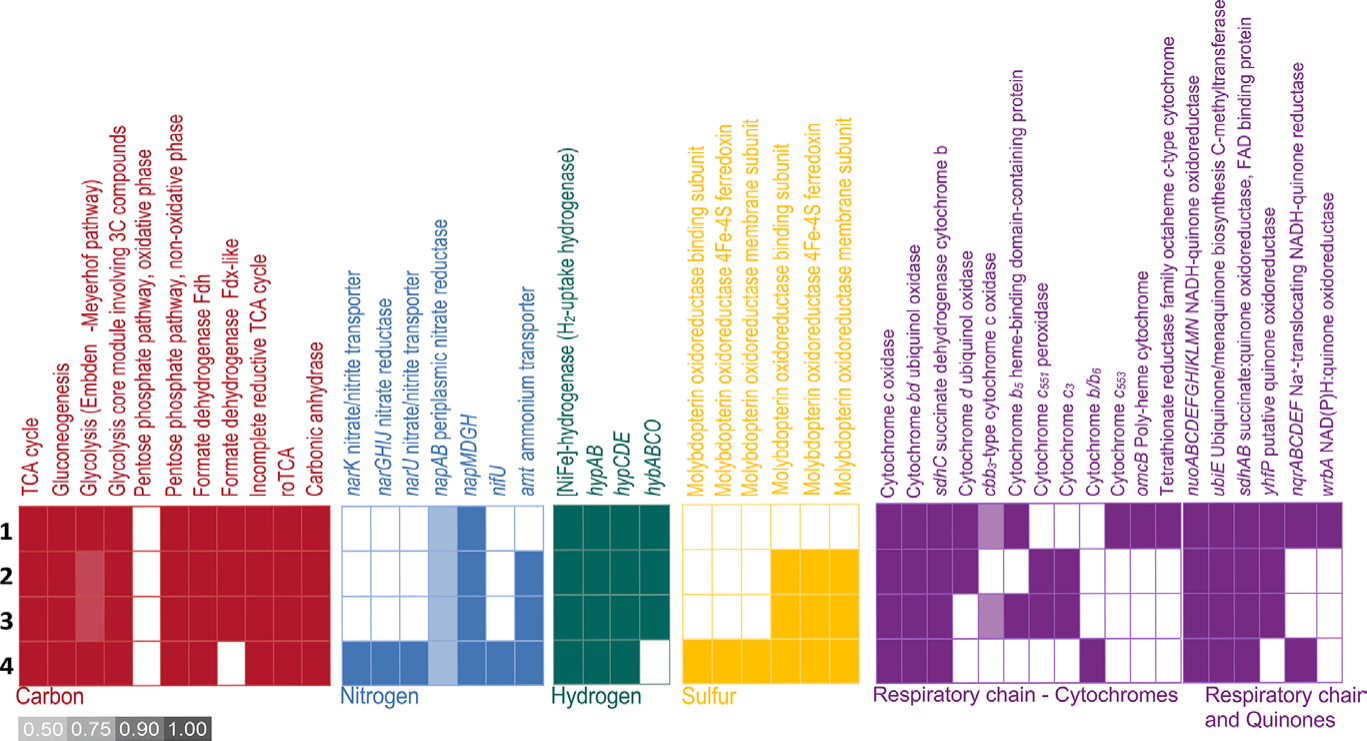
Summary of gene annotations for carbon and energy metabolisms in the four genomes of *Deferribacter* species. 1, *D. thermophilus* BMA^T^; 2, *D. abyssi* JR^T^; 3, *D. autotrophicus* SL50^T^; 4, *D. desulfuricans* SSM1^T^.

It was experimentally demonstrated that the four *Deferribacter* species could use organic acids as energy and carbon source, which is consistent with the genome annotation [26,29]. In fact, all genes encoding the tricarboxylic acid cycle (TCA) were identified, including citrate synthase, aconitate hydratase, isocitrate dehydrogenase, 2-oxoglutarate/2-oxoacid ferredoxin oxidoreductase, succinyl-CoA ligase, succinate dehydrogenase, fumarate hydratase and malate dehydrogenase (locus tags from MaGe are given in Table S3). In addition, the four *Deferribacter* species are capable of oxidizing formate as the sole carbon and energy source and producing CO_2_ through up to two membrane-bound formate dehydrogenase complexes (Fig. 1, Table S3). The first complex was detected in the four *Deferribacter* species (DEFABY_v1_80092–94; DEFTHER_v1_60045_47; FHQ18_v1_50199–201 and DEFDS_1329–31), and the second was only detected in *D. abyssi*, *D. thermophilus* and *D. autotrophicus* (DEFABY_v1_50042–43; DEFTHER_v1_110065–66 and FHQ18_v1_60193–94) (Fig. 1).

Unlike the genomes of *D. desulfuricans* and *D. thermophilus*, those of *D. abyssi* and *D. autotrophicus* did not encode for the complete Embden–Meyerhof–Parnas (EMP) pathway due to the lack of a glucokinase, enabling the phosphorylation of ∝-d-glucose to produce ∝-d-glucose-6-phosphate. However, those latter genomes encoded for a phosphoglucomutase (DEFABY_v1_70034 and FHQ18_v1_50038) that could integrate ∝-d-glucose-1-phosphate resulting from starch and sucrose metabolism into the glycolysis pathway (Fig. 1). The four genomes encoded the full set of genes of the non-oxidative branch of the pentose phosphate pathway (PP) (Fig. 1, Table S3). As mentioned by Slobodkin *et al*. [25], the EMP and PP pathways could probably operate in the reverse direction and could be involved in the synthesis of cellular material during autotrophic growth of *Deferribacter* species.

As the autotrophic growth of both *D. abyssi* and *D. autotrophicus* was experimentally demonstrated and confirmed by *in silico* features for *D. autotrophicus*, with the presence of five genes of the reverse oxidative TCA (roTCA) cycle [25], this pathway for inorganic carbon dioxide fixation has also been investigated here. The four genomes encoded all genes involved in the reverse TCA cycle but did not encode the ATP-citrate lyase. However, as already suggested for *D. autotrophicus*, these strains could use the roTCA cycle thanks to the citrate synthase (CS) operating in reverse direction instead of the ATP citrate lyase, under chemolithoautotrophic conditions [25]. In this case, the CS could cleave citrate adenosine triphosphate independently into acetyl-CoA and oxaloacetate [54]. Interestingly, the CS was identified in the four genomes (Table S3). In addition, all genomes encoded anaplerotic enzymes, such as the phosphoenolpyruvate carboxykinase (EC: 4.1.1.49; DEFABY_v1_80037, DETHER_v1_140024, FHQ18_v1_50245, DEFDS_1461), the pyruvate carboxylase (EC: 6.4.1.1; DEFABY_v1_150014, DETHER_v1_800074 and DEFTHER_v1_60136, FHQ18_v1_10114, DEFDS_1275) and the malate dehydrogenase (EC: 1.1.1.37; DEFABY_v1_190023, DEFTHER_v1_10209, FHQ18_v1_50008, DEFDS_1074). Those enzymes replenish the TCA intermediates that are necessary for biomass synthesis, thereby maintaining their availability in the cycle. In view of these observations, and according to Slobodkin *et al*. [25], it would appear that the four species belonging to the *Deferribacter* genus can grow under autotrophic conditions (with CO_2_ as their sole carbon source). However, although autotrophic growth was demonstrated experimentally for *D. autotrophicus* and *D. abyssi*, this functional feature needs to be confirmed for *D. thermophilus* and *D. desulfuricans*.

### Dissimilatory nitrogen metabolism

Several species isolated from DSHV, including members of the *Deferribacteres* class, such as *Deferribacter abyssi* [27], as well as *Campylobacteria* [55,56], such as *Nautilia nitratireducens* [57], and oilfield-associated bacteria such as *Pseudomonas*, *Geobacillus* or *Marinobacter* [58], have demonstrated their ability to reduce nitrate. This reduction occurs either to nitrite via denitrification or to ammonium through the dissimilatory nitrate reduction to ammonium process (DNRA). While the first step of the DNRA, the nitrate reduction to nitrite, is well known, the second step, the reduction of nitrite to ammonium, primarily involving the NrfA multiheme cytochrome *c*, varies among organisms [59]. For each *Deferribacter* species, the nitrate reduction to nitrite was experimentally evidenced, while the ammonium production was only demonstrated for *D. autotrophicus* [29].

To date, it is established that the dissimilatory nitrate reduction to nitrite can occur by two different systems in bacteria, either using the respiratory membrane-bound Nar-type nitrate reductase or the dissimilatory periplasmic Nap-type nitrate reductase [60,62]. Bacteria can possess either the Nar-type or the Nap-type system, but some species can harbour both, as previously evidenced in *D. desulfuricans* [63]. While the Nar-type system was only detected in the genome of *D. desulfuricans*, the Nap-type system was identified in the four genomes of *Deferribacter* species (Fig. 1). Although the Nap-system consists of two subunits, the catalytic subunit (NapA) and the electron transfer subunit (NapB), the *napB* gene was not evidenced in the genomes of the present study nor in the genome of *D. desulfuricans* as previously demonstrated by Takaki *et al*. [63]. These results suggest that the nitrate reductase in *Deferribacter* species is probably monomeric (*i.e*., NapB-independent as observed for *D. desulfuricans* [64]) in contrast to what is typically observed in bacteria (*i.e*., NapB-dependent). In addition, the complete Nap-system, encoded by the *napMADGH* operon, was detected in the four genomes of *Deferribacter* species (locus tags are given in Table S4). It includes the periplasmic multiheme *c*-type cytochrome NapM, the periplasmic nitrate reductase subunit NapA, the chaperone NapD and the ferredoxin-containing electron transfer subunits NapG and NapH.

The Nap-type system can also be coupled to the reduction of nitrite to either ammonium or nitrogen. To date, the reduction of nitrite to ammonium had only been evidenced in *D. autotrophicus* [26,29]. Nonetheless, Slobodkin *et al*. [25] suggested that the second step of the DNRA could be carried out via the reversible action of a hydroxylamine oxidoreductase, as previously demonstrated in *Nautilia profundicola* [65]. Interestingly, this enzyme was encoded in synteny with the Nap-type system in the genomes of *D. abyssi*, *D. thermophilus* and *D. autotrophicus* (locus tags are given in Table S4) and could be involved in the second DNRA step. Concerning *D. desulfuricans*, a sequence with 89.1% similarity to the hydroxylamine oxidoreductase gene encoded in the genome of *D. abyssi* (DEFABY_v1_50069) was identified with MaGe and annotated as a multiheme *c*-type cytochrome. However, alignment using SwissProt showed a similarity of this enzyme with the hydroxylamine oxidoreductase of *Kuenenia stuttgartiensis* (25.9%), underlining its possible role in the second DNRA step.

### Hydrogen metabolism

It was experimentally demonstrated that the four *Deferribacter* species were able to use hydrogen as an energy source [26,29]. The hydrogen oxidation, coupled to elemental sulphur or Fe(III)-reduction, could be an alternative strategy for energy conservation in this genus. In the present study, a [NiFe]-hydrogenase was detected in all *Deferribacter* genomes. This membrane-bound [NiFe]-hydrogenase, encoded by *hyd* and *hyo* genes, was previously identified in *D. desulfuricans* and *D. autotrophicus*, respectively [25,63], and was also identified in the genomes of *D. abyssi* and *D. thermophilus* (locus tags are given in Table S4). This membrane-bound [NiFe]-hydrogenase could function as a H_2_-uptake hydrogenase where the oxidation of molecular hydrogen is coupled to the reduction of menaquinone. It could have different roles such as in *Geobacter sulfurreducens*, where the hydrogenase is used for oxidative stress defence [66], or, as suggested by Slobodkin *et al*. [25], it could be involved in hydrogenotrophic respiration of elemental sulphur or nitrate. In addition, the genes *hypABCDEF* encoding for the [NiFe]-hydrogenase maturation system were found in the four genomes (locus tags are given in Table S4). Finally, similar to what has been shown for *D. autotrophicus* [25], a five-gene cluster was identified in the genomes of *D. abyssi* and *D. thermophilus* (locus tags are given in Table S4). This cluster contains genes encoding hydrogenase maturation protease and the four [NiFe]-hydrogenase subunits (HybA, HybB, HybC and HybO) forming the membrane-bound [NiFe]-hydrogenase with a periplasmic active site. This complex allows the periplasmic H_2_ oxidation coupled to the reduction of the inner membrane quinone pool, and it belongs to the group 1 [NiFe]-hydrogenase class, involved in H_2_-uptake coupled to terminal electron acceptors, such as metals [67]. Interestingly, the [NiFe]-hydrogenase encoded by the *hybABCO* operon, which could be used for Fe(III)-reduction [25], was not evidenced in the genome of *D. desulfuricans*, the only *Deferribacter* species where the reduction of iron was not experimentally demonstrated. In addition, to reinforce this hypothesis, the operon was identified, using MaGe, in genomes of closely related Fe(III)-reducing bacteria belonging to the *Deferribacterales* order, such as *Deferrivibrio essentukiensis*, *Geovibrio ferrireducens* or *Limisalsivibrio acetivorans*. A non-complete operon has also been found in the genome of the non-Fe(III)-reducing bacterium *Calditerrivibrio nitroreducen*s. Functional analyses will be needed to confirm that the [NiFe]-hydrogenase encoded by the *hybABCO* operon is only used in combination with a metal terminal electron acceptor such as Fe(III) for *D. autotrophicus*, *D. abyssi* and *D. thermophilus*.

### Sulphur metabolism

Deep-sea hydrothermal vents and petroleum reservoirs represent singular environments associated with a high diversity of microorganisms capable of using sulphur compounds for their energetic metabolism. *D. abyssi* has been shown to use elemental sulphur as an electron acceptor, while *D. thermophilus* is unable to use any form of reduced sulphur species as a terminal electron acceptor (Table 1). Sulphur respiration can occur thanks to polysulphide reductase enzymes (PsrABC) either directly or indirectly via soluble polysulphide intermediates [68]. However, discriminating these molybdopterin oxidoreductase enzymes from other molybdopterin oxidoreductases based on sequence alone remains challenging [69]. In the present study, two clusters of three molybdopterin oxidoreductase genes, which have already been identified in *D. autotrophicus* and *D. desulfuricans* genomes, have also been identified in the genome of *D. abyssi* (Fig. 1; locus tags are given in Table S4). These two clusters were not encoded in the genome of *D. thermophilus*, which is unable to respire sulphur compounds (Fig. 1, Table 1). In addition, while those two three-gene clusters of molybdopterin oxidoreductase were not clearly annotated (as tetrathionate or polysulphide reductase), those genes could be involved in the reduction of sulphur compounds. The other enzyme systems, known to be involved in sulphur respiration, HydDACB, ShyCBDA/SuDH, Npsr or SreABC, were not identified [70]. Finally, a molybdopterin oxidoreductase binding subunit was also detected in the *D. thermophilus* genome (DEFTHER_v1_90011), but it was not part of a molybdopterin oxidoreductase cluster. The synteny of this gene showed that it was close to the *sfrB* gene coding also for a NADPH-Fe^3+^ oxidoreductase subunit beta (DEFTHER_v1_90010). Alignment using SwissProt evidenced a similarity of this enzyme with the NADPH-Fe^3+^ oxidoreductase subunit alpha, encoded by the *sfrA* gene (29.5% of similarity). Even though it was hypothesized that the SfrAB complex was involved in the cytoplasmic Fe(III)-reduction in *G. sulfurreducens*, it was demonstrated that it is at work in the acetate metabolism and participates in the NADP regeneration [71].

Finally, no sulphate-reduction enzymes (Sat, AprAB, DsrABC, DsrMKJOP and QmoABC), no sulphide/sulphur oxidation enzymes (Sox system; FccAB; SoeABC; SorAB; Sqr) and no putative gene markers for sulphur disproportionation *(i.e.*, *yedE*, *tusA* and *dsrC*) were identified in all *Deferribacter* genomes [70,72].

### Respiratory chain

In the *Deferribacterales* order, the energy production is mainly performed by oxidative phosphorylation, so by the oxidation of electron donors through an electron transport chain. The respiratory chain is composed of several membrane-bound multiprotein complexes that couple electron transport to the build-up of an electrochemical proton gradient. A first entry point for electrons is the reduction of respiratory quinones, especially by the coenzyme NADH. As already evidenced for *Deferribacterales*, notably in the *Denitrovibrio* family, this step could be performed by two complexes. These complexes are either the proton-pumping NADH-quinone oxidoreductases encoded by 14-genes containing the *nuo*-cluster (*nuoA-N*) or the Na^+^-translocating NADH-quinone reductase encoded by the six genes *nqr*-cluster (*nqrA-F*) [73]. While the *nuo*-cluster was entirely encoded in the four *Deferribacter* genomes (locus tags are given in Table S4), the *nqr*-cluster was only present in the genomes of *D. thermophilus* (DEFTHER_v1_100030–35) and *D. desulfuricans* (Fig. 1, Table S4). In addition, all genomes of *Deferribacter* possessed the complete succinate-quinone dehydrogenase complex, involved in the energy metabolism coupled to the TCA cycle. Finally, the key enzyme, C-methyltransferase encoded by the *ubiE* gene, involved in the carbon methylation for the biosynthesis of ubiquinone (coenzyme Q) or menaquinone (vitamin K2), was detected in all four genomes (Fig. 1, Table S4). This enzyme was shown to be essential for the anaerobic respiration of nitrate [74]. Furthermore, the reducing equivalents essential to nitrate or polysulphide reduction could be provided by NADH quinone oxidoreductases *(i.e*., *nuo* or *nqr* cluster) or succinate/quinone oxidoreductase. Interestingly, the *yhfP* gene, coding for a putative NADP-dependent quinone oxidoreductase, was only detected in the genomes of *D. abyssi*, *D. thermophilus* and *D. autotrophicus*, which are all capable of using Fe(III) as an electron acceptor (Fig. 1). The role of this protein in their metabolism would benefit from further study.

In addition, a variety of cytochromes were detected in all four *Deferribacter* genomes, including the haem-only cytochrome *bd* ubiquinol oxidase (Fig. 1, Table S4), which could be associated with microoxic respiration (which has never been tested with *Deferribacter* species) [75], resulting in tolerance to low O_2_ concentrations. The cytochrome *bd* ubiquinol oxidase was already evidenced in the strict anaerobe *Moorella thermoacetica* to be involved in oxidative stress protection and to contribute to a limited tolerance to O_2_ [76]. This is consistent with the environmental distribution of *Deferribacter* species, as revealed by the Sandpiper analysis: 18 occurrences corresponded to hydrothermal vent fields, 15 to oilfields or oil-polluted environments and 5 to the deep-sea plastisphere (Table S5). This distribution aligns with the isolation of strains from DSHV ecosystems, which are reduced systems in contact with surrounding oxic seawater, thereby creating microoxic niches. In complementary ways, the presence of genes coping with oxidative stress offers a significant ecological advantage for these strains evolving in such contrasting environments. Indeed, under oxidative stress conditions, intracellular free Fe(II) can react with H_2_O_2_ through the Fenton reaction, generating highly reactive hydroxyl radicals, known for their damaging effects on cellular components [77]. In the genomes of *D. abyssi* and *D. autotrophicus*, the cytochrome *c*_551_ peroxidase could limit this reaction (Fig. 1, Table S4). Furthermore, in the four *Deferribacter* genomes, a gene encoding rubrerythrin was detected, including one at the end of the *nuo*-cluster (Fig. 1, Table S4). This protein is known to be involved in H_2_O_2_ detoxification via H_2_O reduction [78].

The *cbb_3_*-type cytochrome, from the haem-copper oxidase superfamily and presenting a high affinity for O_2_ [79], was only partially encoded in *D. autotrophicus* and *D. thermophilus* genomes (DEFTHER_v1_60070–71, FHQ18_v1_90174–75) (Fig. 1). Indeed, the genes *ccoN*, coding for the catalytic subunit, and *ccoO*, coding for the mono-haem membrane-bound *c*-type cytochrome, were present, while the genes *ccoP*, coding for the second membrane-bound *c*-type cytochrome, and *ccoQ*, with a still unclear function but essential for cytochrome stability [80], were not evidenced (Fig. 1).

Finally, the gene coding for cytochrome OmcB, an outer-membrane *c*-type polyheme cytochrome, which is known to be involved in Fe(III)-reduction metabolism in *G. sulfurreducens* [18], was identified only in the *D. thermophilus* genome (DEFTHER_v1_40177), by HMM (Hidden Markov model) profiles using the software FeGenie and with the MaGe platform (Fig. 1). SwissProt alignment confirmed the annotation as OmcB. This gene also appeared to be part of a three-gene cluster that also included genes coding for a *c*-type cytochrome (DEFTHER_v1_40178) and a protein of unknown function (DEFTHER_v1_40179). Alignment of this protein with the UniProt database provided evidence that this last gene encoded a porin. Those three genes thus composed a porin-cytochrome protein complex (PCC), identified firstly in *S. oneidensis* MR-1 with the *mtr* genes [81,82] and then in *G. sulfurreducens* PCA with *omc* genes [83], showing that PCC is a common mechanism for transmembrane electron transfer.

### Iron metabolism

**Fig. 2.**
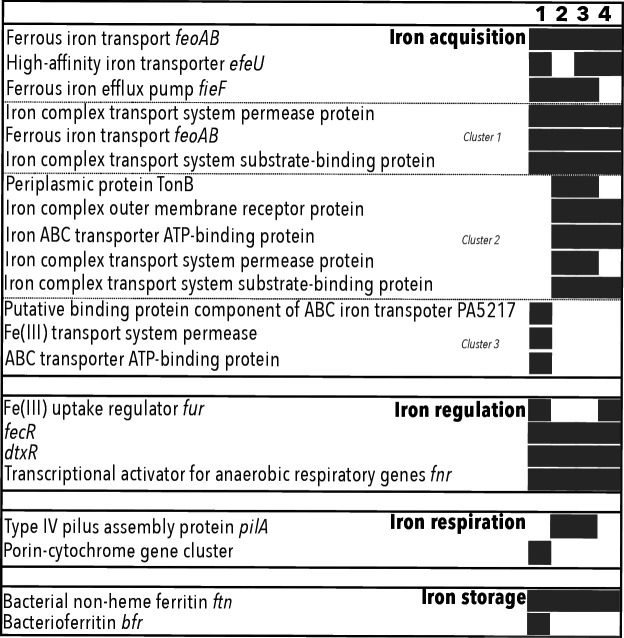
Summary of genes involved in the iron metabolism of the four *Deferribacter* species, predicted using MaGe and/or FeGenie. 1, *D. thermophilus* BMA^T^; 2, *D. abyssi* JR^T^; 3, *D. autotrophicus* SL50^T^; 4, *D. desulfuricans* SSM1^T^.

The FeGenie software and MaGe annotations allowed the identification of genes involved in Fe acquisition, regulation, respiration and storage. However, no genes involved in magnetosome formation, iron oxidation or in haem transport were identified (Fig. 2). Concerning Fe acquisition, genes encoding for the Fe(II) transporter complex FeoAB were identified in the four genomes of *Deferribacter* species (DEFABY_v1_50045–46, DEFTHER_v128-29, FHQ18_v1_60197–98 and DEFDS_0436–37). This is consistent with their ability to grow under anaerobic conditions where iron is mainly found in its reduced form (*i.e*., Fe(II)). In addition, the gene encoding the inner transmembrane pore *efeU* was predicted in all genomes, except in the genome of *D. abyssi* (DEFTHER_v1_120051, FHQ18_v1_100087 and DEFDS_0706). It was suggested that EfeU is a permease for both iron forms, Fe(II) and Fe(III), but with a higher affinity for Fe(II). It is part of an operon, which is induced under low pH conditions and includes EfeO and EfeB, two periplasmic iron transporters [84]. However, in *Deferribacter* genomes, these last two proteins were not predicted. These transporters are used by bacteria to maintain their iron homeostasis and to respond to their iron cellular requirements under low pH and iron-restricted aerobic environments. However, to be functional, the entire *efeUOB* operon is required [84]. The labile intracellular Fe(II) can be used for Fe-responsive gene regulation, such as the Fe(III) uptake regulator *fur*, which was predicted in all four genomes of *Deferribacter* with the FeGenie tool, but not with MaGe (the latter detected the *fur* gene only in *D. desulfuricans* and *D. thermophilus* genomes (DEFDS_1164, DEFTHER_v1_80007). Homologous sequences were detected via MaGe in *D. abyssi* and *D. autotrophicus* (DEFABY_v1_ 220011, FHQ18_v1_80110) with, respectively, 76.1 and 78.4% similarity to the *fur* gene of *D. thermophilus*. These genes were annotated as a peroxide-responsive repressor, PerR. Iron can also be stored in macromolecules such as the non-haem ferritin encoded by the *ftn* gene and identified in all four genomes (DEFABY_v1_20001, DEFTHER_v1_70110, FHQ18_v1_10237 and DEFDS_0829) [85]. Then, Fe could also be involved in redox stress resistance systems. However, as previously stated, an excess of Fe(II) could lead to the Fenton reaction to form reactive oxygen species that can damage proteins, membranes or DNA. That is why export systems such as the Fe(II) efflux pump FieF are necessary and were detected in *D. abyssi*, *D. thermophilus* and *D. autotrophicus* (DEFABY_v1_60060, DEFTHER_v1_30049 and FHQ18_v1_60090) [86]. In addition, in the genome of *D. desulfuricans*, a gene having 78.5% similarity with the *fief* gene was identified (DEFDS_1992) and annotated as a cation efflux protein.

Under iron starvation or because of its low solubility at neutral pH and under anaerobic conditions, bacteria can synthesize organic compounds to transport Fe(III) through the cytoplasmic membrane. These molecules, called siderophores, have a high affinity for Fe(III) [87,88]. In Gram-negative bacteria, such as species of the *Deferribacter* genus, siderophores allow the solubilization of the Fe(III) to shuttle it back to the periplasm thanks to outer membrane transporters, such as TonB-dependent transporters [89,90]. Then, the complex Fe(III)-siderophore is imported across the inner membrane through the ABC transporter associated with periplasmic binding proteins or through proton-motive force-dependent permeases [91]. In the studied genomes, three gene clusters involved in iron acquisition were identified (Fig. 2; locus tags from MaGe are shown in Table S6). The first three-gene cluster (cluster 1 in Fig. 2) was identified in all four genomes of *Deferribacter* species. It was composed of (i) an iron complex transport system permease protein, (ii) an iron ABC transporter ATP-binding protein and (iii) an iron complex transport system substrate-binding protein. The second cluster (cluster 2 in Fig. 2) was only detected in the *Deferribacter* species isolated from DSHV (*i.e. D. abyssi*, *D. autotrophicus* and *D. desulfuricans*), but in *D. desulfuricans*, the cluster was not complete. This cluster was composed of five genes annotated as (i) a periplasmic protein TonB (DEFABY_v1_130048, FHQ18_v1_90061), (ii) an iron complex outer membrane receptor protein (DEFABY_v1_130049, FHQ18_v1_90062, DEFDS_0532), (iii) an iron ABC transporter ATP-binding protein (DEFABY_v1_130051, FHQ18_v1_90063, DEFDS_0536), (iv) an iron complex transport system permease (DEFABY_v1_130052, FHQ18_v1_90064, DEFDS_0532) and (v) an iron complex transport system substrate-binding protein (DEFABY_v1_130053, FHQ18_v1_90065, DEFDS_0533). The SwissProt alignments have shown several matches for the majority of the proteins cited above, but similarity percentages were too low (<50%) to ascertain a protein identity (Table S6). Nonetheless, it could be noticed that several of these proteins could be involved in the transport of the vitamin B_12_ (or cobalamin), the petrobactin, the Fe(III)-hydroxamate or the Fe(III)-citrate (Table S6). While no genes involved in the hydroxamate or petrobactin synthesis have been identified, the complete anaerobic biosynthetic pathway of vitamin B_12_ was encoded in the genome of *D. desulfuricans*, while it was incomplete in the three other genomes. Overall, insoluble Fe(III) transport may be mediated by the vitamin B_12_ transport system for *D. desulfuricans*, but it is still unclear in the other three species. Finally, the last cluster (cluster 3 in Fig. 2) was identified only in the genome of *D. thermophilus* and included three genes annotated as (i) a putative binding protein component of ABC iron transport (DEFTHER_v1_140008), (ii) an Fe(III) transport system permease (DEFTHER_v1_140009) and (iii) an ABC transporter ATP-binding protein (DEFTHER_v1_140010). The SwissProt alignments indicated that these genes corresponded to an iron uptake protein FutA1 (46% similarity), a Fe(III)-transport system permease protein FbpB (24.9%) and a Fe(III) ion import ATP-binding protein FbpC (40.1%), respectively. The FutA1 protein is known to bind Fe(II) to protect the cell against oxidative stress, but also to bind the Fe(III) in the cyanobacterium *Synechocystis* sp. strain PCC 6803 [92].

**Fig. 3.**
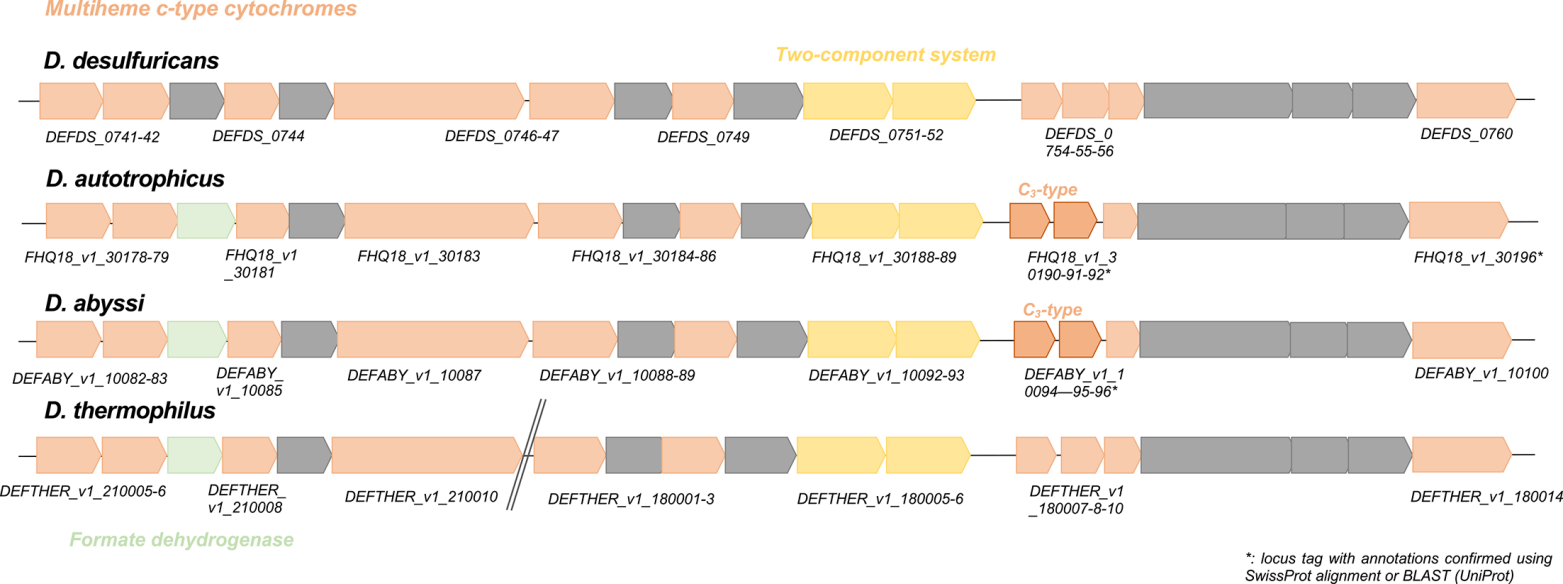
Genomic organization of the 19-gene cluster probably involved in Fe(III)-reduction and detected in the genomes of the four *Deferribacter* species. Legend: in light green, gene coding for the formate dehydrogenase; in light orange, gene encoding cytochromes; in yellow, gene encoding two-component systems; and in grey, other genes.

Genes involved in iron respiration were predicted by the FeGenie software and the MaGe pipeline. The *pilA* gene, encoding the type IV pilus assembly protein, involved in Fe(III)-insoluble reduction, was identified in the genomes of *D. abyssi* (DEFABY_v1_40047) and *D. autotrophicus* (FHQ18_v1_30320). In the present study, a *pilA* gene was also identified in *Geothermobacter ehrlichii*, a Fe(III)-reducer isolated from DSHV. This sequence shared 76.6% similarity to the *pilA* sequence of *D. abyssi*. Interestingly, it has been demonstrated that when the *pilA* gene is deleted from the genome of *G. metallireducens*, the pili production fails, and the mutant strain is no longer able to reduce crystalline Fe(III) oxides, highlighting the importance of this gene towards the respiration of Fe(III) [93]. Indeed, *pilA* is responsible for the production of ‘e-pilin’ or ‘nanowires’ that can be essential for EET, from the cell wall to the mineral [94]. Electrically conductive pili provide a flexible and extended electronic network that expands the prospects for electrical contact with small oxide minerals [95]. However, PilA proteins are widespread in *Bacteria*, and their conductive property is attributed to their specific composition in AAAs, as previously described in *G. metallireducens* and *G. sulfurreducens* [96,97]. Sequence alignment of the PilA sequences from these two *Geobacter* species, along with those detected in *D. abyssi* and *D. autotrophicus*, revealed very similar sequences with a high content in AAAs (Fig. S1). Indeed, the number of AAAs in PilA sequences ranged from 6 (*G. sulfureducens*) to 8 (*G. metallireducens*). AAAs at positions 9 (F), 32 (F), 35 (Y) and 65 (Y) were conserved among the 4 PilA sequences. *D. abyssi* and *D. autotrophicus* also possessed AAAs that were unique to them but were shared with at least one species of *Geobacter* (Fig. S1). These results, showing the high density of AAAs in PilA sequences of *D. abyssi* and *D. autotrophicus*, support the hypothesis that their pili are conductive and facilitate EET for Fe(III) respiration. The presence of a gene encoding *pilA* in *D. desulfuricans* was previously evidenced [25]; however, in the present study, this gene (DEFDS_1270) was annotated as a hypothetical protein (75.8% similarity with the *pilA* sequence of *D. abyssi*). Finally, three genes involved in the respiration of Fe(III) were highlighted in the genome of *D. thermophilus* and corresponded to (i) the *c*-type polyheme cytochrome OmcB (DEFTHER_v1_40177), (ii) a cytochrome *c* (DEFTHER_v1_40178) and (iii) a protein of unknown function (DEFTHER_v1_40179) (Fig. 2). This three-gene porin-cytochrome cluster described in *Geobacter* species is directly involved in EET, allowing the growth under anaerobic conditions by respiring insoluble metal oxides [83,98]. Previous results have demonstrated the impact of those gene deletions on the capacity of *G. metallireducens* to reduce Fe(III)-citrate or Fe(III)-oxides [99]. Moreover, a 19-gene cluster containing many genes encoding membrane-bound (inner or outer membrane) or periplasmic multiheme *c*-type cytochromes has been identified in the genome of *D. desulfuricans* [63]. Based on analyses performed with *G. sulfurreducens* and *S. oneidensis*, which have shown the potential involvement of a multiheme cytochrome in the dissimilatory reduction of metal ions [100,101], it was suggested that this cluster may be a remnant of the Fe(III)-reduction system in *D. desulfuricans* [63]. This cluster was also identified in the genomes of *D. autotrophicus* [25] and here in the genome of *D. abyssi* and *D. thermophilus* (Fig. 3). In the genome of the latter, this cluster was divided into two distinct parts (DEFTHER_v1_180001–14 and DEFTHER_v1_210005–10) (Fig. 3). In the genomes of the two deep-sea hydrothermal strains, *D. abyssi* and *D. autotrophicus*, the 19-gene cluster was highly conserved, sharing between 91.3 and 99.5% of gene similarity (Table S7). Interestingly, two cytochromes *c_3_* were present in this 19-gene cluster in the genomes of *D. abyssi* and *D. autotrophicus* (DEFABY_v1_10094–95, FHQ18_v1_30190–91). These cytochromes have been demonstrated to be involved in Fe(III) respiration in *Shewanella frigidimarina* [102]. Finally, a formate dehydrogenase was identified at the third position in this gene cluster in the genomes of all three Fe(III)-reducing species (*i.e*., *D. abyssi*, *D. autotrophicus* and *D. thermophilus*). Fe(III)-reduction coupled to formate oxidation is a common metabolism in surface and subsurface sediments and in anaerobic environments [103]. In *D. desulfuricans*, a gene with 80.1% similarity with the formate dehydrogenase identified in *D. abyssi* was detected and annotated as a hypothetical protein (DEFDS_0743). The UniProt alignment of this gene showed 100% similarity with a cytochrome *b_561_*. This cytochrome, widespread in the living world, is a two-haem cytochrome involved in transmembrane electron transport and could operate as a Fe(III)-reductase [101,104]. Interestingly, two other genes coding for cytochrome *c_3_* were present in *D. abyssi* and *D. autotrophicus* genomes, outside this 19-gene cluster. One gene (DEFABY_v1_70024 and FHQ18_v1_50050) had homologous sequences annotated as multiheme cytochromes in *D. thermophilus* (DEFTHER_v1_200016) and *D. desulfuricans* (DEFDS_0821), but for the other genes (DEFABY_v1_50072 and FHQ18_v1_60225), no homologous sequence was detected in *D. thermophilus* or *D. desulfuricans*. Finally, when the total number of multiheme cytochromes was compared to the total number of *c*-type cytochromes within the four *Deferribacter* species, it was found to range from 81.8% (*D. thermophilus*) to 86.7% (*D. autotrophicus*), showing the high prevalence of multiheme cytochromes across the *Deferribacter* genus (Table S8). Interestingly, these multiheme cytochromes were highly represented in the 19-gene cluster, representing up to 76.9% in the *D. autotrophicus* genome. Furthermore, the highest prevalence was obtained for the two DSHV strains where Fe(III)-reduction is actively performed (Table S8). Values obtained for *D. desulfuricans*, which are close to the ones obtained for *D. thermophilus*, could again be explained by the presence of a remnant of the metal reduction system in the genome of this strain [63].

### Phylogenomics

**Fig. 4.**
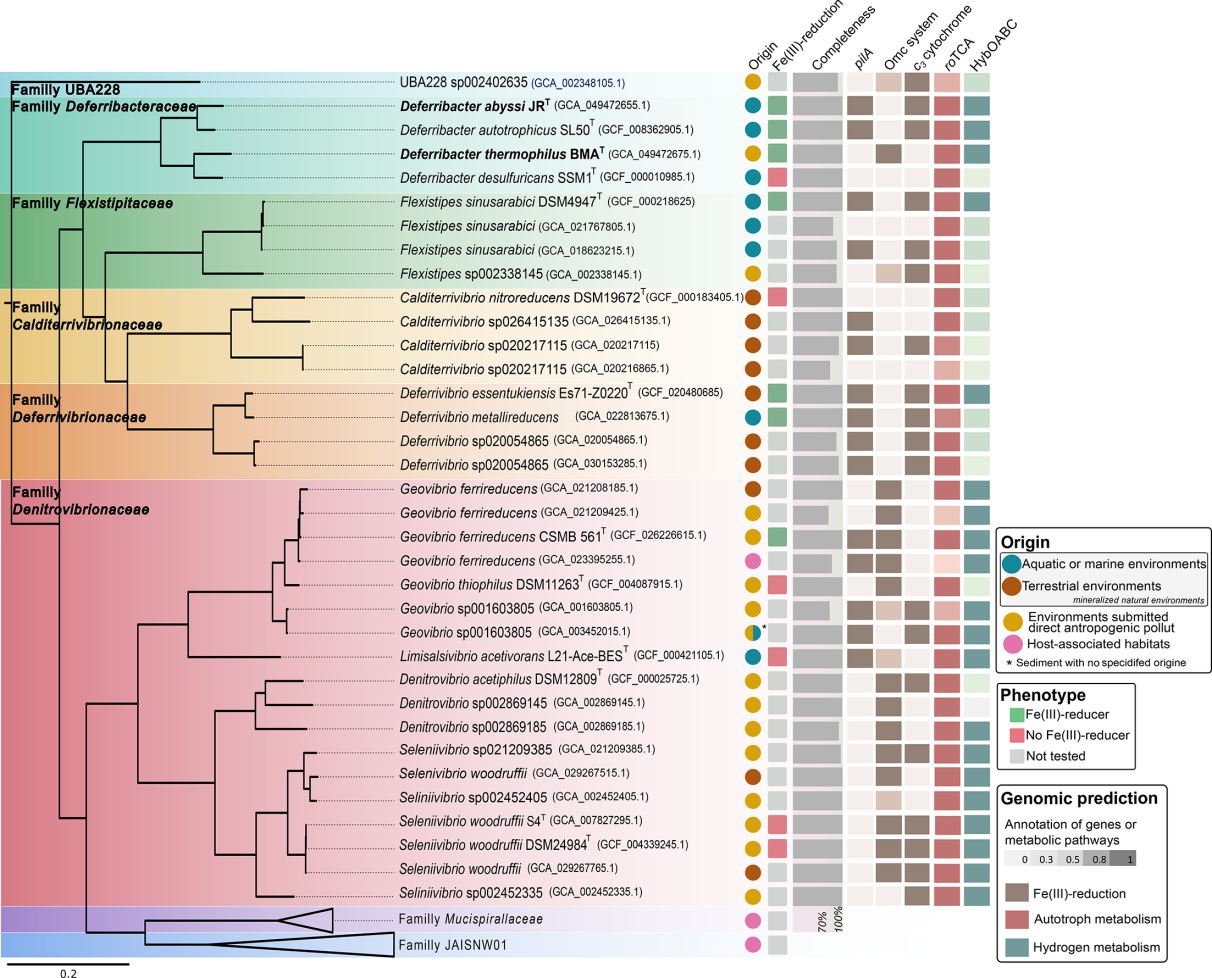
Phylogenomic tree showing the phylogenomic relationship between the *Deferribacter* species and the other representative genomes of type strains or MAGs belonging to the *Deferribacterales* order as well as the origin, the phenotype of isolated strains concerning the Fe(III)-reduction and gene annotations concerning the Fe(III)-reduction (*pilA*, Omc system and *c*_3_ cytochrome), the carbon fixation cycle and the hydrogen metabolism that could be associated with the Fe(III)-reduction. The genome sequence of *Clostridium butyricum* strain CDC51208 was used as an outgroup. The tree was built using IQ-TREE (v. 2.0.3) with the maximum-likelihood method. Bar, 0.2 substitutions per position. The phenotype concerning Fe(III)-reduction was obtained from [26,29,73, 105] Gene annotation was performed using the FeGenie software and the MaGe platform.

To go further into the Fe(III)-reduction metabolism, this study was extended to the *Deferribacterales* order. A phylogenomic analysis was performed based on 14 genomes from type species (including the four *Deferribacter* species) and 36 MAGs of high and medium quality belonging all to the *Deferribacterales* order (Table S1). Those genomes and MAGs display an average length of 2.42 Mbp±0.50 Mbp. Eight clades were obtained in the phylogenomic tree, which corresponded to the eight families of the *Deferribacterales* order described in the GTDB classification (Fig. 4). These families are the following: *Deferribacteraceae*, *Flexistipitaceae*, *Calditerrivibrionaceae*, *Deferrivibrionaceae*, *Denitrovibrionaceae*, *Mucispirallaceae* and the candidate families UBA228 and JAISNW01. The *Denitrovibrionaceae* family referenced in the GTDB database is referenced as *Geovibrionaceae* in the NCBI database [73].

The candidate family UBA228 was the most phylogenetically distant from the other families and was represented by only one high-quality MAG (GCA_002348105.1) obtained from a tailing pond polluted with hydrocarbons. Two main clades were also evidenced and could be distinguished by the origin of the type strains or MAGs (Fig. 4). The first clade was composed of genomes of type strains and MAGs coming from mineralized natural environments and belonging to the *Deferribacteraceae* family, *Flexistipitaceae*, *Calditerrivibrionaceae* and *Deferrivibrionaceae*. Indeed, the two monophyletic groups, comprising genomes of type strains and MAGs of the *Calditerrivibrionaceae* and *Deferrivibrionaceae* families, came from terrestrial environments, whereas the members of *Deferribacteraceae* and *Flexistipitaceae* were mainly derived from marine habitats, except one sequence in each family. Indeed, the genome of the type strain *D. thermophilus* and the MAG representative of the candidate species *Flexistipes* sp002338145 were both obtained from hydrocarbon samples. The second clade was composed of members of the *Denitrovibrionaceae* and *Mucispirallaceae* families and of the candidate family JAISNW01 that are all originating from anthropized or enriched in hydrocarbons environments or gut samples. Indeed, genomes and MAGs of *Denitrovibrionaceae* were derived from locations submitted to anthropogenic pollution and those of the two other families were derived from gut or faeces samples. An exception was evidenced by the type species *Limisalsivibrio acetivorans* L21-Ace-BES^T^, which was isolated from a hypersaline microbial mat [73]. Overall, the heatmap showing the AAI (threshold of 65% for the genus; 95–96% for the species) and ANI (threshold of 95% for the species) values between genomes and MAGs belonging to the order of *Deferribacterales* confirmed the different clusters observed on the phylogenomic tree (Fig. S2).

Concerning their metabolism, all members of the *Deferribacterales* order have the genetic potential to fix CO_2_ through the roTCA, as it was evidenced in all four *Deferribacter* species (Fig. 4). Indeed, the majority of genomes and MAGs encoded for the complete rTCA, except the citrate lyase enzyme, which is replaced by the CS in a majority of genomes and MAGs, which allows the roTCA. It is important to note that the MAGs where the roTCA was not fully detected were also the least complete [completeness <80% for *Calditerrivibrio* sp020217115 (GCA_030153285), *Geovibrio ferrireducens* (GCA_021209425; GCA_023395255) and *Geovibrio* sp001603805 and completeness <93% for UBA228 and *Calditerrivibrio* sp020217115 (GCA_020217115)]. The presence of a complete roTCA cycle in 80% of genomes and MAGs of the *Deferribacterales* order suggests that members of this order could be well involved in primary production processes in diverse environments. Regarding the [NiFe]-hydrogenase encoded by the *hybABCO* operon, which could be used for Fe(III)-reduction, an important number of genomes and MAGs encompassed the complete cluster (Fig. 4). Indeed, 57.1% of genomes and MAGs annotated encoded for the complete *hybABCO* operon (31.3% in the ‘mineralized natural environments’ clade and 83.3% in the ‘anthropized or hydrocarbon-rich environments’ clade) which could be used for Fe(III)-reduction [25].

**Fig. 5.**
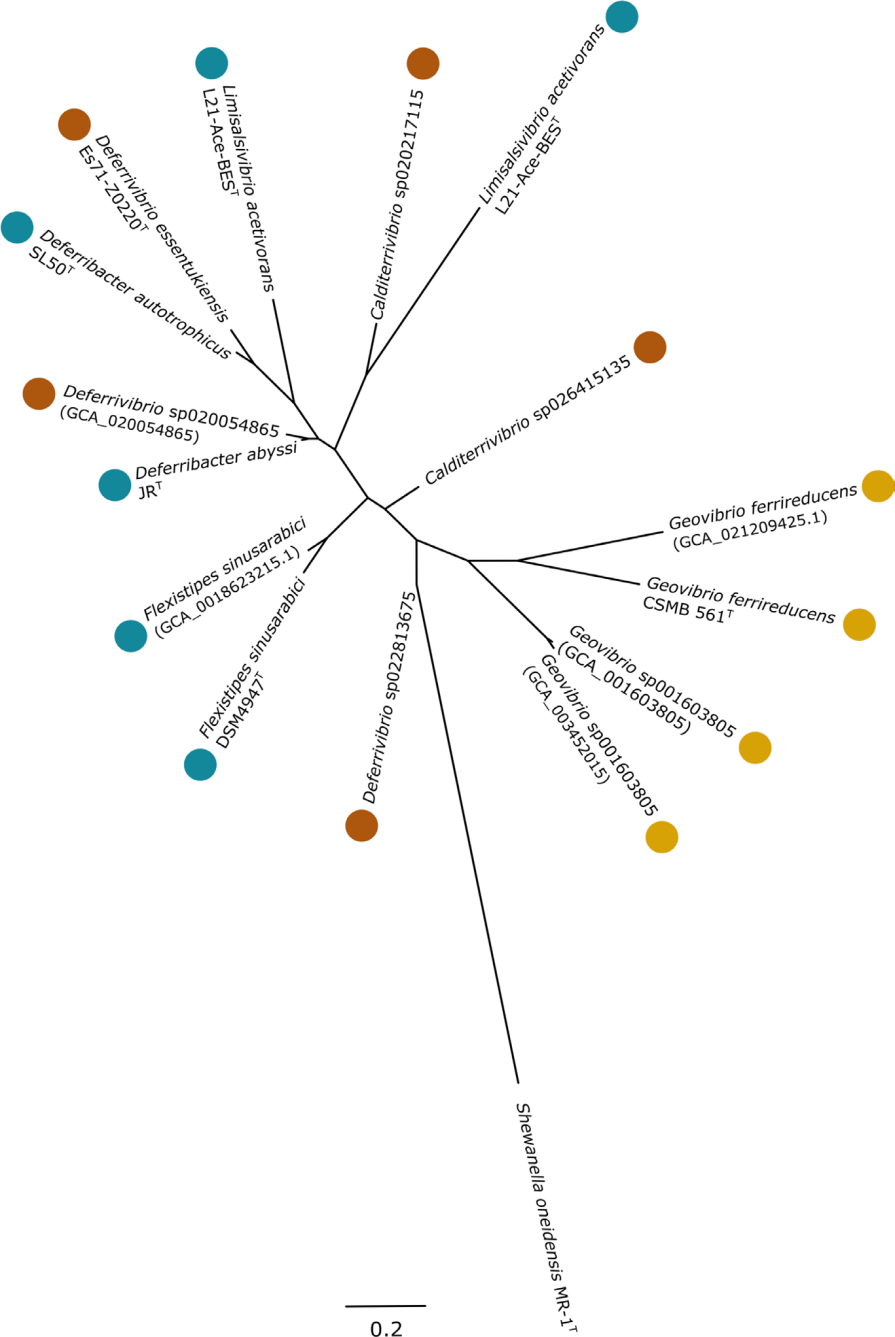
Maximum-likelihood tree of PilA proteins, built with all sequences from genomes and MAGs belonging to the *Deferribacterales* order. The protein sequence of PilA of *Shewanella oneidensis* MR-1 was used as an outgroup. Colour dots indicate the origin of strains or MAGs as in Fig. 4.

With regard to dissimilatory Fe(III)-reduction, a majority of genomes and MAGs encoded for at least one marker gene of Fe(III)-reduction (*i.e*., *pilA* or Omc system (PCC)) or for a gene encoding for the cytochrome *c_3_* involved in Fe(III) respiration in *S. frigidimarina* [102]. Two main systems were identified in genomes and MAGs belonging to the *Deferribacterales* order. Firstly, the Omc system was detected mainly in genomes and MAGs coming from anthropized environments or environments enriched in hydrocarbons (at least one gene of this three-gene cluster was detected in 88.9% against 18.8% in the ‘mineralized natural environments’ clade) (Fig. 4). This system was also encoded in the genome of *D. thermophilus* and in the MAG representing the *Flexistipes* sp002338145 which were also obtained from petroleum well and crude oil, respectively. On the other hand, the gene marker of the Fe(III)-reduction *pilA* was detected in the two clades identified in the phylogenomic tree of the *Deferribacterales* order, but mainly in the ‘mineralized natural environments’ clade. In fact, *pilA* gene was detected twice as much in this clade than in the ‘anthropized or hydrocarbon-rich environments’ clade (62.5% against 27.8%) (Fig. 4). The phylogenetic analysis carried out with all the sequences of this gene revealed a clustering according to the environmental origin of the genomes and MAGs (Fig. 5). Indeed, the *pilA* gene sequences of *Geovibrio* species formed a separate cluster, while the two *pilA* gene sequences detected in the genome of *Limisalsivibrio acetivorans* L21-Ace-BEST, belonging to the same family as *Geovibrio* sp., were part of the cluster formed by *pilA* gene sequences belonging to genomes and MAGs from ‘mineralized natural environments’ (Fig. 5). This result could suggest that the protein involved in Fe(III)-reduction could be subjected to horizontal gene transfer and its own evolution within these two distinct environments. In addition, in the ‘mineralized natural environments’ clade, all Fe(III)-reducing strains encode both the *pilA* and the *c_3_* cytochrome. This finding suggests that, in these environments, the molecular Fe(III)-reducing mechanism may involve these two proteins (Fig. 5). Finally, a notable pattern was observed when considering the percentage of multiheme cytochromes relative to the total number of *c*-type cytochromes in the genomes of *Deferribacterales* type strains, both with experimentally confirmed Fe(III)-reducing capabilities and those without (Table S9). For Fe(III)-reducing species, this percentage ranged from 70.93 to 86.67%. In contrast, non-Fe(III)-reducing species exhibited a lower range of 50–69.57%, except *D. desulfuricans* (82.62%), which may mark a retention of a remnant metal reduction system as previously stated. Consequently, the higher ratio of multiheme cytochromes in Fe(III)-reducing strains could suggest a potential link between the prevalence of multiheme cytochromes and the capacity for Fe(III)-reduction.

Overall, the co-occurrence of genes coding for CO_2_ fixation (roTCA cycle), H_2_ oxidation (protein complex Hyb) and Fe(III)-reduction (PilA or Omc systems) suggests that members of *Deferribacterales* may be able to grow under chemolithoautotrophic conditions consistent with the anoxic and/or iron-rich environments where they have been isolated (*i.e*., hydrothermal systems, sediments, hot springs, subsurface and active sludges).

## Conclusion

**Fig. 6.**
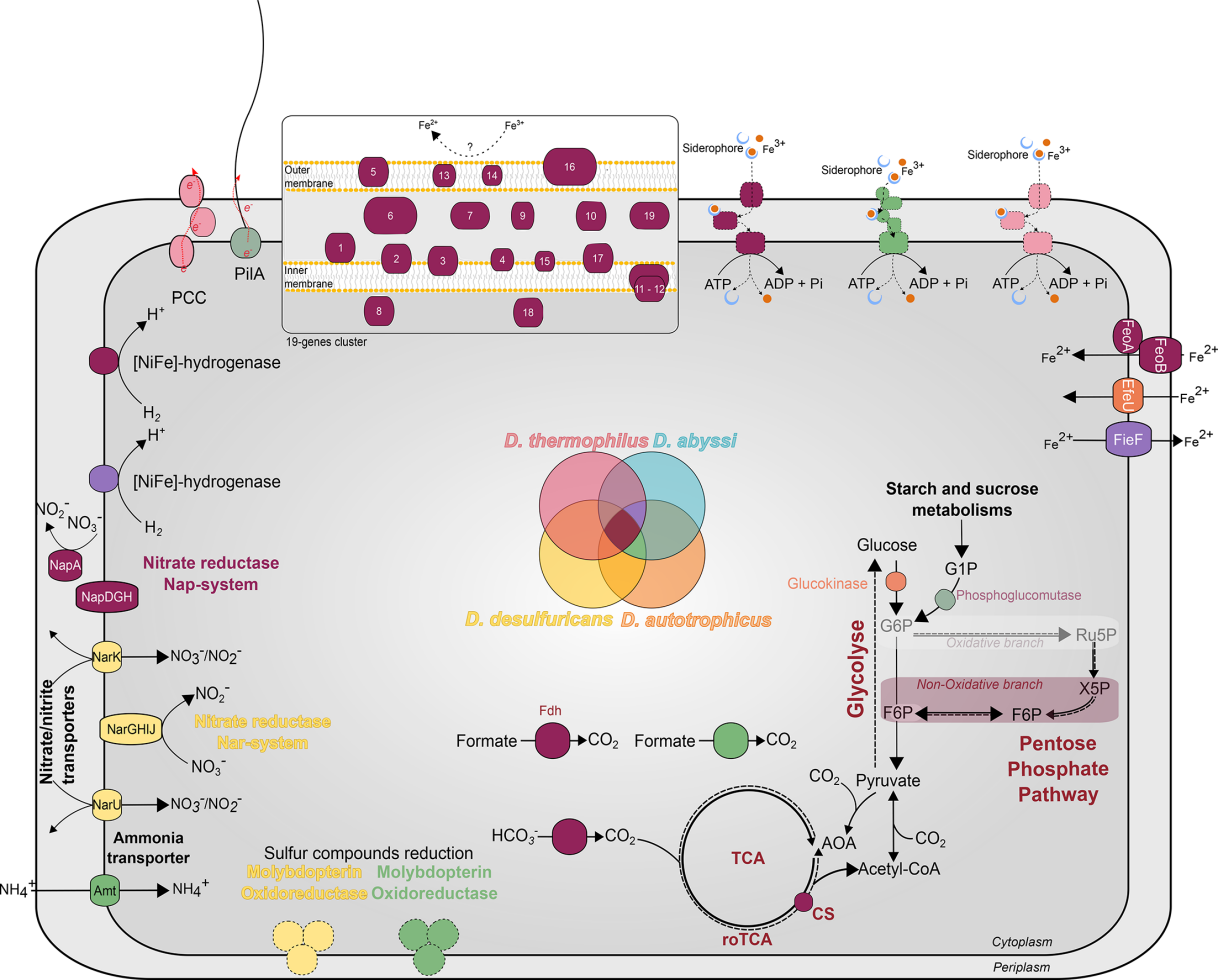
Cell cartoon showing the carbon and energy metabolism of the four *Deferribacter* species. The schematic representation of the 19-gene cluster was modified from Takaki *et al*. [63]. The colour of each protein complex or pathway corresponding to the colour shown in the Venn diagram illustrates their presence in one genome, two genomes or more.

Comparative genomic analysis of the genomes of the four *Deferribacter* species revealed consistency in carbon metabolism, notably in the ability of these species to utilize organic acids via the TCA cycle or to fix CO_2_ as a carbon source via the roTCA cycle (Fig. 6). However, with regard to energy metabolism, some real distinctions were highlighted. In fact, different electron donors and acceptors were identified in each strain (Fig. 6). In addition, the ability of those strains to use different energy and carbon sources illustrates their capacity to adapt to different ecological niches or to persist in highly contrasting niches such as DSHV. With regard to Fe(III)-reduction metabolism in *Deferribacter* species, there appear to be two possible mechanisms. The first is a short-distance mechanism involving a porin-cytochrome protein complex with *omc* genes identified in *D. thermophilus*. The second is a long-distance mechanism involving the pilin *pilA* protein, a key component of electrically conductive nanowires, which was found in *D. abyssi* and *D. autotrophicus*, and to a lesser extent in *D. desulfuricans*. However, the precise mechanisms involved in both cases are still unclear. Both mechanisms have been identified in most genomes and MAGs of *Deferribacterales*, and they appear to be subject to environmental pressure, since the first mechanism has only been detected in genomes and MAGs from anthropized environments or environments enriched in hydrocarbons, whereas the second has been detected preferentially in mineralized natural environments.

## Supplementary material

10.1099/mgen.0.001712 Uncited Supplementary Material 1.

10.1099/mgen.0.001712 Uncited Supplementary Material 2.

## Abbreviations

AAA: aromatic amino acid
AAI: average amino acid identity
ANI: average nucleotide identity
CDS: coding DNA sequences
COG: clusters of orthologous groups
CS: citrate synthase
DNRA: dissimilatory nitrate reduction to ammonium process
DSHV: deep-sea hydrothermal vents
EET: extracellular electron transfer
EMP: Embden–Meyerhof–Parnas
GTDB: Genome Taxonomy Database
MaGe: MicroScope Microbial Genome Annotation and Analysis Platform
MAGs: metagenome-assembled genomes
PCC: porin-cytochrome protein complex
PGAP: Prokaryotic Genome Annotation Pipeline
PP: pentose phosphate pathway
roTCA: reverse oxidative TCA
SCGs: single-copy core genes
TCA: tricarboxylic acid cycle

## References

[1] Kendall B, Anbar AD, Kappler A, Konhauser KO (2012). Fundamentals of Geobiology.

[2] Tagliabue A, Bopp L, Dutay J-C, Bowie AR, Chever F (2010). Hydrothermal contribution to the oceanic dissolved iron inventory. Nature Geosci.

[3] Tagliabue A, Resing J (2016). Impact of hydrothermalism on the ocean iron cycle. Phil Trans R Soc A.

[4] Emerson D (2015). The irony of iron - biogenic iron oxides as an iron source to the ocean. Front Microbiol.

[5] Bonnet S, Guieu C, Taillandier V, Boulart C, Bouruet-Aubertot P (2023). Natural iron fertilization by shallow hydrothermal sources fuels diazotroph blooms in the ocean. Science.

[6] Feely RA, Lewison M, Massoth GJ, Robert‐Baldo G, Lavelle JW (1987). Composition and dissolution of black smoker particulates from active vents on the Juan de Fuca Ridge. J Geophys Res.

[7] Fitzsimmons JN, John SG, Marsay CM, Hoffman CL, Nicholas SL (2017). Iron persistence in a distal hydrothermal plume supported by dissolved–particulate exchange. Nature Geosci.

[8] Colombo C, Palumbo G, He JZ, Pinton R, Cesco S (2014). Review on iron availability in soil: interaction of Fe minerals, plants, and microbes. J Soils Sediments.

[9] Li M, Toner BM, Baker BJ, Breier JA, Sheik CS (2014). Microbial iron uptake as a mechanism for dispersing iron from deep-sea hydrothermal vents. Nat Commun.

[10] Shi L, Richardson DJ, Wang Z, Kerisit SN, Rosso KM (2009). The roles of outer membrane cytochromes of *Shewanella* and *Geobacter* in extracellular electron transfer. Environ Microbiol Rep.

[11] Manzella MP, Holmes DE, Rocheleau JM, Chung A, Reguera G (2015). The complete genome sequence and emendation of the hyperthermophilic, obligate iron-reducing archaeon “*Geoglobus ahangari*” strain 234(T). Stand Genomic Sci.

[12] Kashyap S, Holden JF (2021). Microbe–mineral interaction and novel proteins for iron oxide mineral reduction in the hyperthermophilic crenarchaeon *Pyrodictium delaneyi*. Appl Environ Microbiol.

[13] Pankratova G, Hederstedt L, Gorton L (2019). Extracellular electron transfer features of Gram-positive bacteria. Anal Chim Acta.

[14] Liu J, Yang S, Mehta N, Deng H, Jiang Y (2025). Alkane degradation coupled to Fe(III) reduction mediated by Gram-positive bacteria. J Hazard Mat.

[15] Bird LJ, Bonnefoy V, Newman DK (2011). Bioenergetic challenges of microbial iron metabolisms. Trends Microbiol.

[16] Schicklberger M, Sturm G, Gescher J (2013). Genomic plasticity enables a secondary electron transport pathway in *Shewanella oneidensis*. Appl Environ Microbiol.

[17] Shi L, Rosso KM, Zachara JM, Fredrickson JK (2012). Mtr extracellular electron-transfer pathways in Fe(III)-reducing or Fe(II)-oxidizing bacteria: a genomic perspective. *Biochem Soc Trans*.

[18] Leang C, Coppi MV, Lovley DR (2003). OmcB, a c-type polyheme cytochrome, involved in Fe(III) reduction in *Geobacter sulfurreducens*. J Bacteriol.

[19] Leang C, Lovley DR (2005). Regulation of two highly similar genes, omcB and omcC, in a 10 kb chromosomal duplication in *Geobacter sulfurreducens*. Microbiology.

[20] Kim B-C, Leang C, Ding Y-HR, Glaven RH, Coppi MV (2005). OmcF, a putative c-type monoheme outer membrane cytochrome required for the expression of other outer membrane cytochromes in Geobacter sulfurreducens. J Bacteriol.

[21] Inoue K, Qian X, Morgado L, Kim B-C, Mester T (2010). Purification and characterization of OmcZ, an outer-surface, octaheme c-type cytochrome essential for optimal current production by Geobacter sulfurreducens. Appl Environ Microbiol.

[22] Qian X, Mester T, Morgado L, Arakawa T, Sharma ML (2011). Biochemical characterization of purified OmcS, a c-type cytochrome required for insoluble Fe(III) reduction in *Geobacter sulfurreducens*. *Biochim Biophys Acta*.

[23] McAllister SM, Vandzura R, Keffer JL, Polson SW, Chan CS (2021). Aerobic and anaerobic iron oxidizers together drive denitrification and carbon cycling at marine iron-rich hydrothermal vents. ISME J.

[24] Smith H, Abuyen K, Tremblay J, Savalia P, Pérez-Rodríguez I (2018). Genome sequence of *Geothermobacter* sp. strain HR-1, an iron reducer from the Lō’ihi Seamount, Hawai’i. Genome Announc.

[25] Slobodkin A, Slobodkina G, Allioux M, Alain K, Jebbar M (2019). Genomic insights into the carbon and energy metabolism of a thermophilic deep-sea bacterium *Deferribacter autotrophicus* revealed new metabolic traits in the phylum *Deferribacteres*. Genes.

[26] Greene AC, Patel BKC, Sheehy AJ (1997). *Deferribacter thermophilus* gen. nov., sp. nov., a novel thermophilic manganese-and iron-reducing bacterium isolated from a petroleum reservoir. Int J Syst Evol Microbiol.

[27] Miroshnichenko ML, Slobodkin AI, Kostrikina NA, L’Haridon S, Nercessian O (2003). *Deferribacter abyssi* sp. nov., an anaerobic thermophile from deep-sea hydrothermal vents of the Mid-Atlantic Ridge. Int J Syst Evol Microbiol.

[28] Takai K, Kobayashi H, Nealson KH, Horikoshi K (2003). *Deferribacter desulfuricans* sp. nov., a novel sulfur-, nitrate- and arsenate-reducing thermophile isolated from a deep-sea hydrothermal vent. Int J Syst Evol Microbiol.

[29] Slobodkina GB, Kolganova TV, Chernyh NA, Querellou J, Bonch-Osmolovskaya EA (2009). *Deferribacter autotrophicus* sp. nov., an iron(III)-reducing bacterium from a deep-sea hydrothermal vent. Int J Syst Evol Microbiol.

[30] Charbonnier F, Forterre P, Erauso G, Prieur D (1995). Archaea, a Laboratory Manual, Thermophiles.

[31] Bolger AM, Lohse M, Usadel B (2014). Trimmomatic: a flexible trimmer for Illumina sequence data. Bioinformatics.

[32] Bankevich A, Nurk S, Antipov D, Gurevich AA, Dvorkin M (2012). SPAdes: a new genome assembly algorithm and its applications to single-cell sequencing. J Comput Biol.

[33] Seemann T (2017). Shovill: faster spades assembly of illumina reads.

[34] Gurevich A, Saveliev V, Vyahhi N, Tesler G (2013). QUAST: quality assessment tool for genome assemblies. Bioinformatics.

[35] Parks DH, Imelfort M, Skennerton CT, Hugenholtz P, Tyson GW (2015). CheckM: assessing the quality of microbial genomes recovered from isolates, single cells, and metagenomes. Genome Res.

[36] Vallenet D, Calteau A, Dubois M, Amours P, Bazin A (2020). MicroScope: an integrated platform for the annotation and exploration of microbial gene functions through genomic, pangenomic and metabolic comparative analysis. Nucleic Acids Res.

[37] Chklovski A, Parks DH, Woodcroft BJ, Tyson GW (2023). CheckM2: a rapid, scalable and accurate tool for assessing microbial genome quality using machine learning. Nat Methods.

[38] Seemann T (2014). Prokka: rapid prokaryotic genome annotation. Bioinformatics.

[39] Wee SK, Yap EPH (2021). GALAXY workflow for bacterial next-generation sequencing *de novo* assembly and annotation. Curr Protoc.

[40] Tatusova T, DiCuccio M, Badretdin A, Chetvernin V, Nawrocki EP (2016). NCBI prokaryotic genome annotation pipeline. Nucleic Acids Res.

[41] Huerta-Cepas J, Forslund K, Coelho LP, Szklarczyk D, Jensen LJ (2017). Fast genome-wide functional annotation through orthology assignment by eggNOG-mapper. Mol Biol Evol.

[42] Garber AI, Nealson KH, Okamoto A, McAllister SM, Chan CS (2020). FeGenie: a comprehensive tool for the identification of iron genes and iron gene neighborhoods in genome and metagenome assemblies. Front Microbiol.

[43] Boeckmann B, Blatter MC, Famiglietti L, Hinz U, Lane L (2005). Protein variety and functional diversity: Swiss-Prot annotation in its biological context. C R Biol.

[44] Consortium U (2019). UniProt: a worldwide hub of protein knowledge. Nucleic Acids Res.

[45] Woodcroft BJ, Aroney STN, Zhao R, Cunningham M, Mitchell JAM (2025). Comprehensive taxonomic identification of microbial species in metagenomic data using SingleM and Sandpiper. Nat Biotechnol.

[46] Bowers RM, Kyrpides NC, Stepanauskas R, Harmon-Smith M, Doud D (2017). Minimum information about a single amplified genome (MISAG) and a metagenome-assembled genome (MIMAG) of bacteria and archaea. Nat Biotechnol.

[47] Chaumeil PA, Mussig AJ, Hugenholtz P, Parks DH (2020). GTDB-Tk: a toolkit to classify genomes with the Genome Taxonomy Database. *Bioinformatics*.

[48] Capella-Gutiérrez S, Silla-Martínez JM, Gabaldón T (2009). trimAl: a tool for automated alignment trimming in large-scale phylogenetic analyses. Bioinformatics.

[49] Minh BQ, Schmidt HA, Chernomor O, Schrempf D, Woodhams MD (2020). IQ-TREE 2: new models and efficient methods for phylogenetic inference in the genomic era. Mol Biol Evol.

[50] Rambaut A, Drummond AJ (2012). FigTree version 1.4.0.

[51] Jain C, Rodriguez-R LM, Phillippy AM, Konstantinidis KT, Aluru S (2018). High throughput ANI analysis of 90K prokaryotic genomes reveals clear species boundaries. Nat Commun.

[52] Kim D, Park S, Chun J (2021). Introducing EzAAI: a pipeline for high throughput calculations of prokaryotic average amino acid identity. *J Microbiol*.

[53] Larkin MA, Blackshields G, Brown NP, Chenna R, McGettigan PA (2007). Clustal W and Clustal X version 2.0. Bioinformatics.

[54] Mall A, Sobotta J, Huber C, Tschirner C, Kowarschik S (2018). Reversibility of citrate synthase allows autotrophic growth of a thermophilic bacterium. Science.

[55] Nakagawa S, Takai K, Inagaki F, Horikoshi K, Sako Y (2005). *Nitratiruptor tergarcus* gen. nov., sp. nov. and *Nitratifractor salsuginis* gen. nov., sp. nov., nitrate-reducing chemolithoautotrophs of the epsilon-Proteobacteria isolated from a deep-sea hydrothermal system in the Mid-Okinawa Trough. Int J Syst Evol Microbiol.

[56] Takai K, Suzuki M, Nakagawa S, Miyazaki M, Suzuki Y (2006). *Sulfurimonas paralvinellae* sp. nov., a novel mesophilic, hydrogen- and sulfur-oxidizing chemolithoautotroph within the Epsilonproteobacteria isolated from a deep-sea hydrothermal vent polychaete nest, reclassification of *Thiomicrospira denitrificans* as *Sulfurimonas denitrificans* comb. nov. and emended description of the genus *Sulfurimonas*. Int J Syst Evol Microbiol.

[57] Pérez-Rodríguez I, Ricci J, Voordeckers JW, Starovoytov V, Vetriani C (2010). *Nautilia nitratireducens* sp. nov., a thermophilic, anaerobic, chemosynthetic, nitrate-ammonifying bacterium isolated from a deep-sea hydrothermal vent. Int J Syst Evol Microbiol.

[58] Semenova EM, Ershov AP, Sokolova DS, Tourova TP, Nazina TN (2020). Diversity and biotechnological potential of nitrate-reducing bacteria from heavy-oil reservoirs (Russia). Microbiology.

[59] Simon J, Klotz MG (2013). Diversity and evolution of bioenergetic systems involved in microbial nitrogen compound transformations. Biochim Biophys Acta.

[60] Moreno-Vivián C, Cabello P, Martínez-Luque M, Blasco R, Castillo F (1999). Prokaryotic nitrate reduction: molecular properties and functional distinction among bacterial nitrate reductases. J Bacteriol.

[61] Philippot L, Højberg O (1999). Dissimilatory nitrate reductases in bacteria. *Biochim Biophys Acta*.

[62] Rusmana I, Nedwell DB (2004). Use of chlorate as a selective inhibitor to distinguish membrane-bound nitrate reductase (Nar) and periplasmic nitrate reductase (Nap) of dissimilative nitrate reducing bacteria in sediment. FEMS Microbiol Ecol.

[63] Takaki Y, Shimamura S, Nakagawa S, Fukuhara Y, Horikawa H (2010). Bacterial lifestyle in a deep-sea hydrothermal vent chimney revealed by the genome sequence of the thermophilic bacterium *Deferribacter desulfuricans* SSM1. DNA Res.

[64] Marietou A, Richardson D, Cole J, Mohan S (2005). Nitrate reduction by *Desulfovibrio desulfuricans*: a periplasmic nitrate reductase system that lacks NapB, but includes a unique tetraheme c-type cytochrome, NapM. FEMS Microbiol Lett.

[65] Hanson TE, Campbell BJ, Kalis KM, Campbell MA, Klotz MG (2013). Nitrate ammonification by *Nautilia profundicola* AmH: experimental evidence consistent with a free hydroxylamine intermediate. Front Microbiol.

[66] Tremblay PL, Lovley DR (2012). Role of the NiFe hydrogenase Hya in oxidative stress defense in *Geobacter sulfurreducens*. J Bacteriol.

[67] Vignais PM, Billoud B, Meyer J (2001). Classification and phylogeny of hydrogenases. FEMS Microbiol Rev.

[68] Hedderich R, Klimmek O, Kröger A, Dirmeier R, Keller M (1998). Anaerobic respiration with elemental sulfur and with disulfides. FEMS Microbiol Rev.

[69] Rothery RA, Workun GJ, Weiner JH (2008). The prokaryotic complex iron–sulfur molybdoenzyme family. *Biochim Biophys Acta*.

[70] Wasmund K, Mußmann M, Loy A (2017). The life sulfuric: microbial ecology of sulfur cycling in marine sediments. Environ Microbiol Rep.

[71] Coppi MV, O’Neil RA, Leang C, Kaufmann F, Methé BA (2007). Involvement of *Geobacter sulfurreducens* SfrAB in acetate metabolism rather than intracellular, respiration-linked Fe(III) citrate reduction. *Microbiology*.

[72] Yvenou S, Allioux M, Slobodkin A, Slobodkina G, Jebbar M (2021). Genetic potential of *Dissulfurimicrobium hydrothermale*, an obligate sulfur-disproportionating thermophilic microorganism. Microorganisms.

[73] Spring S, Rohde M, Bunk B, Spröer C, Will SE (2022). New insights into the energy metabolism and taxonomy of *Deferribacteres* revealed by the characterization of a new isolate from a hypersaline microbial mat. Environ Microbiol.

[74] Lee PT, Hsu AY, Ha HT, Clarke CF (1997). A C-methyltransferase involved in both ubiquinone and menaquinone biosynthesis: isolation and identification of the *Escherichia coli* ubiE gene. J Bacteriol.

[75] Jünemann S (1997). Cytochrome bd terminal oxidase. Biochim Biophys Acta.

[76] Das A, Silaghi-Dumitrescu R, Ljungdahl LG, Kurtz DM (2005). Cytochrome bd oxidase, oxidative stress, and dioxygen tolerance of the strictly anaerobic bacterium *Moorella thermoacetica*. J Bacteriol.

[77] Cohen G (1985). Handbook of Methods for Oxygen Radical Research.

[78] Dillard BD, Demick JM, Adams MWW, Lanzilotta WN (2011). A cryo-crystallographic time course for peroxide reduction by rubrerythrin from *Pyrococcus furiosus*. *J Biol Inorg Chem*.

[79] Pitcher RS, Watmough NJ (2004). The bacterial cytochrome cbb3 oxidases. *Biochim Biophys Acta*.

[80] Peters A, Kulajta C, Pawlik G, Daldal F, Koch HG (2008). Stability of the cbb3-type cytochrome oxidase requires specific CcoQ-CcoP interactions. J Bacteriol.

[81] Hartshorne RS, Reardon CL, Ross D, Nuester J, Clarke TA (2009). Characterization of an electron conduit between bacteria and the extracellular environment. Proc Natl Acad Sci USA.

[82] Richardson DJ, Butt JN, Fredrickson JK, Zachara JM, Shi L (2012). The “porin-cytochrome” model for microbe-to-mineral electron transfer. Mol Microbiol.

[83] Liu Y, Wang Z, Liu J, Levar C, Edwards MJ (2014). A trans-outer membrane porin-cytochrome protein complex for extracellular electron transfer by *Geobacter sulfurreducens* PCA. Environ Microbiol Rep.

[84] Cao J, Woodhall MR, Alvarez J, Cartron ML, Andrews SC (2007). EfeUOB (YcdNOB) is a tripartite, acid-induced and CpxAR-regulated, low-pH Fe2+ transporter that is cryptic in *Escherichia coli* K-12 but functional in *E. coli* O157:H7. Mol Microbiol.

[85] Smith JL (2004). The physiological role of ferritin-like compounds in bacteria. Crit Rev Microbiol.

[86] Grass G, Otto M, Fricke B, Haney CJ, Rensing C (2005). FieF (YiiP) from *Escherichia coli* mediates decreased cellular accumulation of iron and relieves iron stress. Arch Microbiol.

[87] Miethke M, Marahiel MA (2007). Siderophore-based iron acquisition and pathogen control. Microbiol Mol Biol Rev.

[88] Stintzi A, Barnes C, Xu J, Raymond KN (2000). Microbial iron transport via a siderophore shuttle: a membrane ion transport paradigm. Proc Natl Acad Sci USA.

[89] Bradbeer C (1993). The proton motive force drives the outer membrane transport of cobalamin in *Escherichia coli*. J Bacteriol.

[90] Braun V (1995). Energy-coupled transport and signal transduction through the gram-negative outer membrane via TonB-ExbB-ExbD-dependent receptor proteins. FEMS Microbiol Rev.

[91] Faraldo-Gómez JD, Sansom MSP (2003). Acquisition of siderophores in gram-negative bacteria. Nat Rev Mol Cell Biol.

[92] Katoh H, Hagino N, Ogawa T (2001). Iron-binding activity of FutA1 subunit of an ABC-type iron transporter in the cyanobacterium *Synechocystis* sp. Strain PCC 6803. Plant Cell Physiol.

[93] Tremblay PL, Aklujkar M, Leang C, Nevin KP, Lovley D (2012). A genetic system for *Geobacter metallireducens*: role of the flagellin and pilin in the reduction of Fe(III) oxide. Environ Microbiol Rep.

[94] Reguera G, McCarthy KD, Mehta T, Nicoll JS, Tuominen MT (2005). Extracellular electron transfer via microbial nanowires. Nature.

[95] Holmes DE, Dang Y, Walker DJF, Lovley DR (2016). The electrically conductive pili of *Geobacter s*pecies are a recently evolved feature for extracellular electron transfer. Microb Genom.

[96] Vargas M, Malvankar NS, Tremblay P-L, Leang C, Smith JA (2013). Aromatic amino acids required for pili conductivity and long-range extracellular electron transport in *Geobacter sulfurreducens*. mBio.

[97] Tan Y, Adhikari RY, Malvankar NS, Ward JE, Woodard TL (2017). Expressing the *Geobacter metallireducens* PilA in *Geobacter sulfurreducens* yields pili with exceptional conductivity. mBio.

[98] Zhuo S, Jiang Y, Qi L, Hu Y, Jiang Z (2024). The robustness of porin-cytochrome gene clusters from *Geobacter metallireducens* in extracellular electron transfer. mBio.

[99] Smith JA, Lovley DR, Tremblay P-L (2013). Outer cell surface components essential for Fe(III) oxide reduction by *Geobacter metallireducens*. Appl Environ Microbiol.

[100] Shi L, Squier TC, Zachara JM, Fredrickson JK (2007). Respiration of metal (hydr)oxides by *Shewanella* and *Geobacter*: a key role for multihaem c-type cytochromes. Mol Microbiol.

[101] Edwards MJ, Richardson DJ, Paquete CM, Clarke TA (2020). Role of multiheme cytochromes involved in extracellular anaerobic respiration in bacteria. Protein Sci.

[102] Gordon EH, Pike AD, Hill AE, Cuthbertson PM, Chapman SK (2000). Identification and characterization of a novel cytochrome c(3) from *Shewanella frigidimarina* that is involved in Fe(III) respiration. Biochem J.

[103] Lovley DR, Phillips EJ, Lonergan DJ (1989). Hydrogen and formate oxidation coupled to dissimilatory reduction of iron or manganese by *Alteromonas putrefaciens*. Appl Environ Microbiol.

[104] Bérczi A, Zimányi L (2014). The trans-membrane cytochrome b561 proteins: structural information and biological function. Curr Protein Pept Sci.

[105] Fiala G, Woese CR, Langworthy TA, Stetter KO (1990). *Flexistipes sinusarabici*, a novel genus and species of eubacteria occurring in the Atlantis II Deep brines of the Red Sea. Arch Microbiol.

[106] Caccavo Jr. F, Coates JD, Rossello-Mora RA, Ludwig W, Schleifer KH (1996). *Geovibrio ferrireducens*, a phylogenetically distinct dissimilatory Fe(III)-reducing bacterium. Arch Microbiol.

[107] Myhr S, Torsvik T (2000). *Denitrovibrio acetiphilus*, a novel genus and species of dissimilatory nitrate-reducing bacterium isolated from an oil reservoir model column. Int J Syst Evol Microbiol.

[108] Janssen PH, Liesack W, Schink B (2002). *Geovibrio thiophilus* sp. nov., a novel sulfur-reducing bacterium belonging to the phylum *Deferribacteres*. Int J Syst Evol Microbiol.

[109] Iino T, Nakagawa T, Mori K, Harayama S, Suzuki KI (2008). *Calditerrivibrio nitroreducens* gen. nov., sp. nov., a thermophilic, nitrate-reducing bacterium isolated from a terrestrial hot spring in Japan. Int J Syst Evol Microbiol.

[110] Rauschenbach I, Posternak V, Cantarella P, McConnell J, Starovoytov V (2013). *Seleniivibrio woodruffii* gen. nov., sp. nov., a selenate- and arsenate-respiring bacterium in the *Deferribacteraceae*. Int J Syst Evol Microbiol.

[111] Zavarzina DG, Prokofeva MI, Pikhtereva VA, Klyukina AA, Maslov AA (2022). *Deferrivibrio essentukiensis* sp. nov., gen. nov., a representative of *Deferrivibrionaceae* fam. nov., isolated from the subsurface aquifer of Caucasian mineral drinking waters. Microbiology.

[112] Galès G, Hennart M, Hannoun M, Postec A, Erauso G (2025). Metabolic versatility and nitrate reduction pathways of a new thermophilic bacterium of the *Deferrivibrionaceae*: *Deferrivibrio metallireducens* sp. nov isolated from hot sediments of Vulcano Island, Italy. PLoS One.

