# Supplementary material for: Phylogenomic and metabolic insights into iron reduction metabolism in the genus Deferribacter belonging to the order Deferribacterales
Source: Microb Genom. 2026 May 19;12(5):001712. doi: 10.1099/mgen.0.001712 (PMC13186404; doi:10.1099/mgen.0.001712)
Supplement: Uncited Supplementary Material 1. [file mgen-12-01712-s001.pdf]

Supplementary data

Phylogenomic and metabolic insights into iron reduction metabolism in the genus *Deferribacter* belonging to the order *Deferribacterales*.

Eva Pouder, Karine Alain* and Sophie Mieszkin*,†

List of figures:

```
Geobacter_sulfurreducens        --------FTLIELLIVVAIIGILAAIAAPQFSAYRVKAYNSAASSDLRN
Geobacter_metallireducens       --------FTLIELLIVVAIIGILAAIAAPQFAAYRQKAFNSAAESDLKN
Deferribacter_abyssi            MKKTNKKGFTLIELLVVVAIIGILAAIAIPQFAKYRTRAYNSAAQSDLRN
Deferribacter_autotrophicus     MKKTNKKGFTLIELLVVVAIIGILAAIAIPQFAKYRQRAQDSAALSDLKT
                                        ******* ************ ***  **  *  *** ***

Geobacter_sulfurreducens        LKTALESAFADDQTYPPES
Geobacter_metallireducens       TKTNLESYYSEHQFYPN--
Deferribacter_abyssi            VRGALEAYYADTQQYPSNL
Deferribacter_autotrophicus     IQTTAEAYYSEYMHY----
                                     *        *
```

Figure S1. Alignment of amino acid sequence of PilA detected in *G. sulfurereducens*, *G. metallireducens*, *D. abyssi* and *D. autotrophicus*. The aromatic amino acids (F, Phénylalanine ; T, Tyrosine ; H, Histidine) are colored yellow.

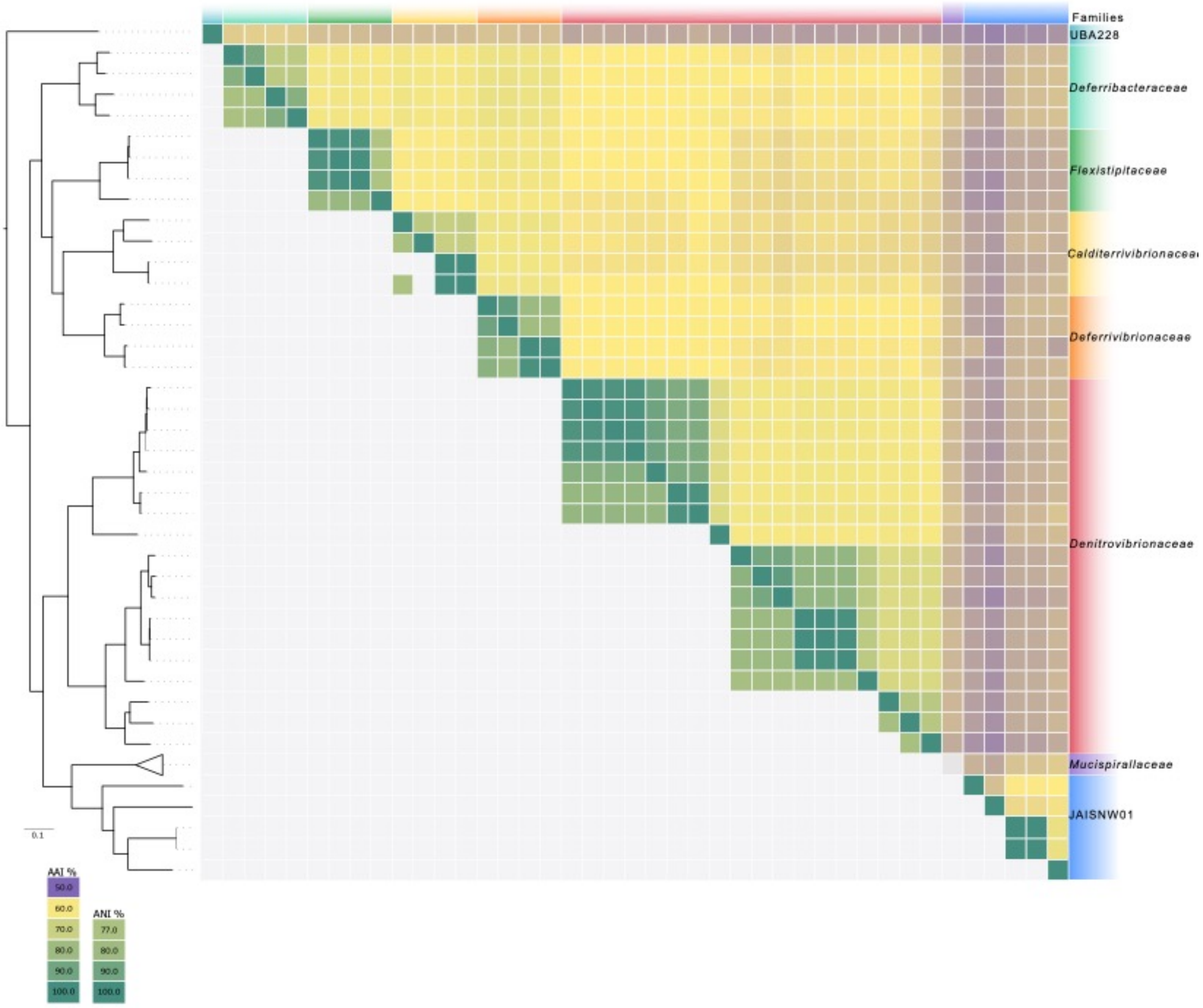

Figure S2. Heatmap of ANI and AAI values calculated using FastANI and EZAAI respectively, between the genomes and MAGs belonging to the order *Deferribacterales*.
